# Shifting From Tokenism to Meaningful Adolescent Participation in Research for Obesity Prevention: A Systematic Scoping Review

**DOI:** 10.3389/fpubh.2021.789535

**Published:** 2021-12-23

**Authors:** Mariam Mandoh, Julie Redfern, Seema Mihrshahi, Hoi Lun Cheng, Philayrath Phongsavan, Stephanie R. Partridge

**Affiliations:** ^1^School of Health Sciences, Faculty of Medicine and Health, The University of Sydney, Sydney, NSW, Australia; ^2^The George Institute for Global Health, The University of New South Wales, Camperdown, NSW, Australia; ^3^Department of Health Systems and Populations, Macquarie University, Sydney, NSW, Australia; ^4^Prevention Research Collaboration, Charles Perkins Centre, Sydney School of Public Health, Faculty of Medicine and Health, The University of Sydney, Camperdown, NSW, Australia; ^5^Sydney Medical School, Faculty of Medicine and Health, Discipline of Child and Adolescent Health, The University of Sydney, Sydney, NSW, Australia; ^6^The Children's Hospital at Westmead, Academic Department of Adolescent Medicine, Sydney, NSW, Australia

**Keywords:** adolescent, youth, participation, engagement, decision-making, obesity, overweight, prevention

## Abstract

**Background:** Traditionally, adolescent participation in research has been tokenistic. Adolescents are rarely afforded the opportunity to influence decision-making in research designed to prevent obesity. Engaging adolescents in meaningful decision-making may enhance research translation. This review aimed to analyze the current modes and nature of adolescent participation in obesity prevention research decision-making.

**Methods:** A systematic scoping review was conducted using Arksey and O'Malley's six-stage framework. Six major databases were searched for peer-reviewed primary research studies with adolescent participation related to obesity, physical activity, and diet. Modes of adolescent participation were categorized based on the Lansdown-UNICEF conceptual framework for measuring outcomes of adolescent participation. The framework outlines three modes of meaningful participation: (i) consultative, which involves taking opinions and needs into consideration; (ii) collaborative, where adolescents are partners in the decision-making process; and (iii) adolescent-led participation where adolescents have the capacity to influence the process and outcomes. The degree of involvement in research cycles was classified based on the National Health and Medical Research Council consumer engagement framework. Five stages of the research cycle were determined: identify, design and develop, conduct, analyze and disseminate.

**Results:** In total, 126 papers describing 71 unique studies were identified. Of these, 69% (49/71) took place in the USA, and 85% (52/61) were conducted in minority or underserved communities, while males were more likely to be under-represented. In 49% (35/71) of studies, participation was consultative and 9% (6/71) of studies involved an adolescent-led approach. Furthermore, 87% (62/71) of studies incorporated adolescent participation in one or more of the research cycle's formative phases, which involve eliciting views, opinions and idea generation. Only 11% of studies engaged adolescents in all five stages of the research cycle where adolescents could have more influence over the research process.

**Conclusion:** Meaningful adolescent participation in the obesity prevention research cycle is limited. Empowering and mobilizing equal partnership with adolescents should be at the forefront of all adolescent-related obesity prevention research.

## Introduction

Overweight and obesity is a global health crisis affecting 340 million young people (5–19 years) worldwide (1). Despite common misconceptions and gender-based stereotypes, obesity is more likely to affect adolescent boys than girls in 60% of countries, with this trend most apparent in high and upper-middle-income nations (2). The prevalence of overweight and obesity in young people continues to increase and has quadrupled in this cohort from 4 to 18% in the last three decades (3). The incidence of overweight and obesity has plateaued in several high-income countries while simultaneously escalating at a 10-fold rate in lower-income countries across Asia and Africa (4). Overweight and obesity are significant risk factors for developing lifestyle-related chronic diseases such as cardiovascular disease, diabetes mellitus, hypertension, some cancers, muscular-skeletal, and mental health disorders (5).

An unhealthy diet and insufficient physical activity are modifiable risk factors for obesity. Social equity challenges, such as income, education, and food insecurity strongly influence these risk factors (6, 7). An increasing body of evidence demonstrates the relationship between diet and physical activity behaviors in adolescence and the association between obesity and chronic disease development in adulthood (8–11). Yet, globally, there is inconsistent evidence regarding the effectiveness of diet or physical activity interventions or a combination of both to reduce the risk of obesity and its related co-morbidities in adolescents (12–14). Obesity prevention research is further complicated by ethical and psychosocial health concerns such as weight stigmatization, eating disorder risk, low self-esteem, body dissatisfaction, anxiety, and depression (15–18). Emerging research demonstrates how supervised obesity treatment can improve psychosocial health for adolescents; however, there is limited research regarding obesity prevention initiatives (19). Engaging adolescents in the design and development of obesity prevention interventions can address social equity challenges and ethical and psychosocial health concerns (20). It is therefore crucial that adolescents from all backgrounds are engaged in obesity prevention research (21).

Current evidence syntheses have focused on determining the efficacy and effectiveness of obesity prevention interventions (12, 14); the conclusions are limited mainly to factors related to intervention outcomes (22). This finding suggests little attention has been given to understanding meaningful adolescent participation in the research decision-making process beyond passive engagement as research participants. Currently, no review has focused on understanding adolescents' engagement in the research cycle and relationships to intervention efficacy and effectiveness.

In recent years, there has been a growing call to prioritize the voice of adolescents in societal decision-making (23). Meaningful adolescent participation is a fundamental human right as articulated in the Convention on the Rights of the Child (24). The importance of adolescent participation is further expanded on in the Lansdown-UNICEF framework for adolescent participation (25). The framework acknowledges that meaningful participation involves adolescents articulating their views and being involved in the decision-making process to impact matters of importance to them. Meaningful participation varies in mode and is subject to the evolving capacity of adolescents as they transition from early adolescence through to young adulthood.

Theoretical youth engagement models such as UNICEFs Adolescent and Youth Engagement Strategic Framework (26), Hart's Ladder (27), and Shier's pathway (28), among others, have been developed to guide organizations on the components of effective and ethical consumer or stakeholder engagement. Such models aim to amplify adolescent voices and improve their confidence and feelings of empowerment, which are fundamental to meaningful participation and decision-making in research and ultimately to research translation (25, 26).

Additionally, practical and transparent frameworks allow researchers to monitor meaningful participation where stakeholders and consumers can be involved effectively throughout the research process. Examples of such are the United Kingdom's National Institute for Health Research (NIHR) INVOLVE Public and Patient Involvement Framework (29, 30), and the Australian National Health and Medical Research Council (NHMRC) Consumer Engagement Framework (31), which provide a detailed outline of the stages and strategies necessary to achieve meaningful participation. Such frameworks offer foundational measures for researchers to assess the degree of engagement expected throughout the research cycle.

Despite the increasing call to effectively engage adolescents in society and consumers in research, there is yet to be meaningful convergence. To date, reviews in the field of participatory research have tended to focus specifically on specific methodological approaches such as Participatory Action Research (PAR) (32, 33), Youth advisory (34), Patient Engagement (PE) (35), or Community Based Participatory Research (CBPR) (36). The demographic of interest also varies significantly between published reviews with researchers specifically investigating children (37) or children grouped with adolescents, youth, or young people and results for such populations analyzed together despite their significantly different needs and abilities (32, 33, 36, 38). Moreover, current evidence syntheses are looking broadly at participatory approaches (32, 33, 36), how they influence social determinants (32), general health and well-being (34, 35, 38), or health policy (39). Currently, comprehensive evidence syntheses are lacking to evaluate the participatory approaches for adolescent consumers in obesity prevention research.

Mental health researchers have recognized that engaging adolescents in meaningful participation is a crucial component of effective research and intervention development (40, 41). Adolescents have unique insight into their own lived experiences and needs; therefore meaningful participation involving adolescent consumers in the decision-making is a potentially vital link to tailor obesity and chronic disease prevention interventions to this demographic. In this scoping review, we aim to address this gap in the literature by broadly assessing how and to what extent adolescents are meaningfully participating in the co-design and decision-making in research studies that target overweight, obesity, physical activity, and dietary interventions specifically for adolescents. Secondly, we aim to provide recommendations on optimal modes of participation in obesity and chronic disease prevention research.

The following research questions guided this scoping review:

i) Is there evidence of the effectiveness of adolescent participation in peer-reviewed primary research studies?
ii) What are the components, processes, or conceptual frameworks of effective peer-reviewed primary research studies involving adolescent participation?
iii) Are there any identified barriers or facilitators, or evidence gaps for adolescent participation?

## Methods

A scoping review was determined to be the most suitable form of evidence synthesis for the research question as it allowed for a broad and thorough examination of the existing literature (42). The scoping review methodology was informed by the Arksey and O'Malley six-stage framework (43), Levac et al.'s (44) recommendations, and the Joanna Briggs Institute (45–47) guidelines for scoping reviews. The review was reported based on the Preferred Reporting Items for Systematic Reviews and Meta-Analyses extension for Scoping Reviews (PRISMA-ScR) checklist (see Supplementary File 1) (48). As per scoping review guidelines, no quality assessment is required (47, 48). The research questions were formulated, followed by the identification of criteria for the inclusion of relevant studies. Next, studies were selected based on the pre-defined criteria, and relevant data from the included studies were extracted. Collating, summarizing, and reporting the results followed. The scoping review protocol contains full details of the study methodology and search strategy (49). The protocol is registered with Joanna Briggs Institute and Open Science Framework, doi: 10.17605/OSF.IO/E3S64. A brief description of the methods is provided below.

### Key Definitions

The term “adolescent” is defined by the World Health Organization (WHO) as a person aged 10–19 years old, and “youth” as one between the age of 15–24 years old (50). However, within the published peer-reviewed literature, these terms are used synonymously. To broadly examine the literature and in line with the Lancet Child and Adolescent health definition of an adolescent (51), this scoping review assessed published peer-reviewed literature engaging adolescents or youth aged 10–24 years.

Meaningful participation refers to participation in which adolescents have some level of influence over the research and development decision-making. The mode of meaningful participation is dependent on the degree of influence adolescents impart on the research process (Table 1). Furthermore, meaningful participation is dependent on the methods and strategies used at various points within the research cycle (31). All modes of participation can be valid and effective if conducted within an enabling environment where participatory outcomes are based on empowerment and the degree of influence is measured and achieved (25).

**Table 1:** Working definitions to classify mode and degree of adolescent participation.

**Mode of adolescent participation**	
Consultative	Adolescents contribute opinions, perspectives, knowledge, and experience
Collaborative	Adolescents are involved as partners in the decision-making process
Adolescent-led	Adolescents identify the issues and control the process and outcomes
Other	Adolescents are involved in an important role, but their opinions are not considered, they play no part in the decision-making and have no influence over the research process or outcomes
**Adolescent participation in stages of the research cycle**	
Identification of topic	The consumers views, opinions or aspirations are sought. Consumers are involved in the identification and development of the research idea and/or topic
Design and Development	The consumer is engaged in methods selection and development
Conduct	Consumers lead or facilitate research methods and gather data
Analyses	Consumers are involved in consolidating and reporting the findings
Dissemination	Consumers are involved in presenting and/ or circulating the findings. Consumers are involved in implementing findings by developing strategies to translate research findings

*Definitions adapted from the Lansdown-UNICEF conceptual framework for measuring outcomes of adolescent participation (25) and Australia's National Health and Medical Research Council (NHMRC) framework for effective consumer and community engagement in research (31)*.

### Search Strategy and Study Selection

Based on key concepts emerging from the research questions, the research team developed a search strategy in consultation with an academic liaison librarian. All qualitative and quantitative peer-reviewed primary research studies published from 1995 to December 2020 were considered for inclusion; reviews were excluded. Peer-reviewed papers of all languages with an abstract in English were considered. Studies involved participants aged 10–24 years involved in obesity or chronic disease prevention, nutrition or physical activity research decision-making. Youth participation was the primary outcome sought hence inclusion of broad search terminologies such as Youth-PAR, CBPR, youth involvement, and youth engagement. Medical subject heading (MeSH) terms were selected accordingly. An example search strategy is presented in Supplementary Table 1.

Systematic searches of six scientific databases, Medline (PubMed), Embase, CINAHL, Scopus, Global health, and CENTRAL, were conducted. Publications identified up to the first of December 2020 were considered. Additional sources were identified through hand searching, reference list examination, and citation chaining. Results were pooled and duplicates removed, remaining results were uploaded to the Covidence systematic review software. A two-part study selection process followed, first a title and abstract review by one reviewer (MM) and secondly a full-text review by two reviewers (MM and SRP). Any discrepancies were resolved by consensus with a third reviewer (JR).

### Data Extraction

Data extraction table variables were iteratively developed to broadly encompass the scope of adolescent participation in the obesity and chronic disease research cycle. Relevant data were extracted and tabulated by one reviewer (MM) (see Supplementary Table 2). Key outcomes extracted included social and environmental variables, the degree of adolescent participation in the research cycle, modes of participation, participatory outcomes, and chronic disease-associated outcomes. Corresponding authors were contacted *via* email where necessary data was missing or not reported. A second reviewer (SRP) cross-checked 20% of studies for consistency in data extraction.

### Synthesis and Analysis of the Results

Data presented were based on active (meaningful) adolescent participation in the research and decision-making process, where adolescents were engaged as consumers rather than passive study participants. Data were assessed based on key modes of participation (consultative, collaborative and, adolescent-led) and alignment with participatory outcomes from the Lansdown-UNICEF conceptual framework for adolescent participation (25). After the protocol publication, the NHMRC framework for effective consumer and community engagement in research was selected to contextualize the data within the phases of the research cycle (31). The framework was deemed necessary for data analysis and was the only protocol deviation.

Chronic disease outcomes associated with improved health status or obesity and chronic disease risk factor prevention were established based on emerging categories founded on preliminary searching. Reported chronic disease outcomes were classified into the following categories: (i) increased awareness of family, peers and adolescents; (ii) program or intervention development; (iii) policy change; (iv) environmental change, relating to changes in the physical surroundings in which adolescents live; (v) behavior change specifically changes in diet and physical activity behaviors; and (iv) health status or risk factor change, including but not limited to change in body mass index, weight status, waist circumference, and blood pressure. Qualitative analysis of the data revealed emerging trends and themes. Data were reported by descriptive numerical analysis and in narrative form.

## Results

### Descriptive Numerical Analysis

**Figure 1:**
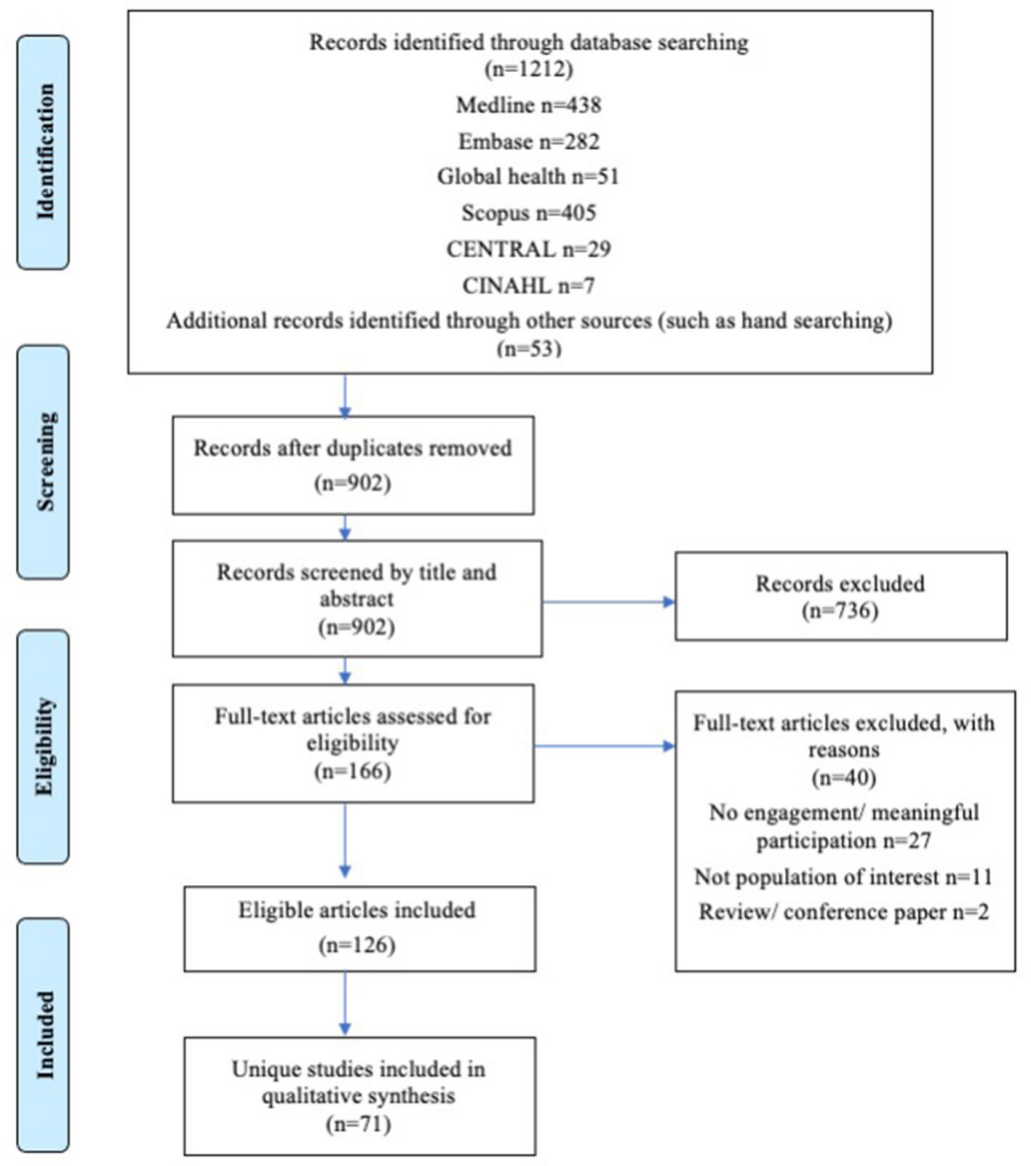
Prisma flow diagram: adolescent participation in research for obesity prevention.

The search strategy generated a total of 1,212 results, including an additional 53 papers through hand searching, which were subsequently pooled, and duplicates removed, leaving 902 unique papers (Figure 1). The 902 papers were assessed by title and abstract screening for inclusion; 736 were excluded. The full text of the remaining 166 papers were reviewed, and a further 40 papers were excluded and reasons documented. In total, 126 full-text peer-reviewed papers (52–175) describing 71 unique studies were included in the review.

### Study Characteristics

The studies identified varied broadly in their target demographic and scope. Various socioeconomic and ecological characteristics were analyzed (Table 2).

**Table 2:** Summary of study characteristics.

	**Sub-category of study characteristics**	***N* (%)**
**Study design** *n* (%)	Mixed methods^*^	21 (30%)
	Qualitative	46 (65%)
	RCT only	4 (6%)
Publication year^**^n (%)	2001–2005	2 (3%)
	2006–2009	8 (11%)
	2010–2014	25 (35%)
	2015–2020	36 (51%)
**Duration of intervention** *n* (%)	≤3 months	23 (37%)
	>3 to <6 months	2 (3%)
	6 to <12 months	7 (11%)
	12 to <18 months	9 (15%)
	18–24 months	9 (15%)
	>24 months	12 (19%)
	Not reported	9 (13%)
**Continent/country** *n* (%)	USA	49 (69%)
	Canada	5 (7%)
	Europe/United Kingdom	8 (11%)
	Other^***^	9 (13%)
**Socio-ecological setting** *n* (%)	Local community *n* (%)	38 (54%)
	Institution's total (schools, hospitals, courts, workplaces) *n* (%)	24 (34%)
	Secondary education *n* (%)	21 (30%)
	Tertiary education	2 (3%)
	Hospital	1 (1%)
	Family	1 (1%)
	Peers^****^ (alone)	0
	Youth/peer leadership/mentoring or education total	18 (25%)
	Local community	6 (8%)
	Institution (secondary school)	12 (18%)
	Youth/peer advocacy/activism total	9 (13%)
	Local community	8 (11%)
	Institution (secondary school)	1 (1%)
	Adolescent (Individual)	8 (11%)
**Main chronic disease or risk factor of interest**	Overweight/obesity	63 (89%)
	Type 2 Diabetes	3 (4%)
	Cardiovascular disease	1 (1%)
	Wellness/determinants of health/NCD^*****^ prevention	3 (4%)
	Physical inactivity	1 (1%)
**Number of participants** *n* (%)	1–10	6 (9%)
	11–50	31 (46%)
	51–100	11 (16%)
	>100	20 (29%)
	Not reported	3 (4%)
	Total number	39079
	Mean	575
	Median	43
	Range	5–14000
**Gender**	Majority female (>60%)	24 (44%)
	Majority male (>60%)	4 (7%)
	Mixed (almost equally)	26 (48%)
	All female	6 (11%)
	All male	2 (4%)
	Not reported	17 (24%)
**Socioeconomic status (SES)/participant representation**	Minority/underserved community/low-income	52 (85%)
	Mixed high and low SES	8 (13%)
	High SES	1 (2%)
	Not reported	10 (14%)

* *Nine studies were RCT's with qualitative components*.

** *Year of publication of the first paper if multiple papers published regarding the same study*.

*** *Five studies in Australia, two in Asia, One in South America and One Multi-country*.

**** *Peer mentoring which took place within an institutional or local community setting was classified under those categories*.

***** *Non-Communicable Disease (NCD)*.

#### Study Design

Of the 71 unique studies identified, 65% (46/71) were qualitative, and a further 30% (21/71) employed a mixed-methods approach. Thirteen studies (18%, 13/71) were randomized controlled trials (RCT).

#### Year of Publication and Study Duration

Publication dates ranged from 2003 to 2020, with >50% (*n* = 36) of studies publishing their first paper between 2015 and 2020. Studies varied in duration from 30-min to 6 years, the mean study duration being 3 months or less in duration (23/71), followed by intervention durations >12 months (12/71).

#### Country of Origin

Most studies (76%, 54/71) were conducted in North America, with a total of 49 in the USA and five in Canada. Eight studies took place in Europe (Netherlands, United Kingdom, Belgium, Spain, France) and five studies in Australia. Furthermore, two studies were conducted in Asia (Republic of Korea, Vietnam), one in South America (Peru), and one multi-country (Australia, New Zealand, Fiji, and Tonga) study.

#### Socioecological Setting

The most common socioecological setting targeted was the local community (54%, 38/71), followed by secondary education institutions (30%, 21/71). Eight studies targeted the adolescent (individual), and one study specifically targeted the family unit. Peer leadership, mentoring, or education were components of 18 studies, with six targeting the local community and 12 targeting secondary education institutions. Similarly, youth or peer advocacy was a component of nine studies, eight of which targeted the local community and one targeting a secondary education institution.

Socioeconomic status (SES) was described in 61/71 studies (86%), of those reporting SES, 52 studies (85%) were conducted in a minority or underserved community, and one study took place in an affluent (high SES) setting.

#### Study Purpose

Overweight and obesity was the most common chronic disease or chronic disease risk factor targeted in the included studies, with 89% (63/71) of studies reporting this to be the main purpose for the study. The remaining eight studies investigated the prevention of obesity-related co-morbidities such as type 2 diabetes and cardiovascular disease.

#### Participants

Of the 71 studies, the total number of participants involved in a meaningful participatory method ranged from 5 to 14,000 participants (Supplementary Table 2), with a median of 43 participants. Thirty-one studies (46%) had between 11 and 50 participants; three studies did not report the number of adolescent participants. The gender of adolescent participants was not reported in 24% of studies (17/71). In 26 (48%) of the studies which reported gender, gender was evenly distributed. Still, in the studies that remained, males were more likely to be under-represented [24 (44%) or not represented at all six (11%), while females were under-represented in four (7%) studies and not represented in a further three (4%)].

### Adolescent Participation

#### Degree and Mode of Participation

In 87% (62/71) of studies, adolescents participated in at least one of the two formative phases of the research cycle (identification of the topic or design or development), and in 11% (8/71) of studies, participation took place in all five stages of the research process (Table 3). The mode of participation was almost equally distributed between consultative (49%, 35/71) and collaborative (41%, 29/71) approaches. An adolescent-led mode of participation was identified in six studies (9%).

**Table 3:** Adolescent participation, theoretical frameworks used and outcomes of participation in the research cycle.

		***N* (%)**
Adolescent participation in the research cycle *n* (%)	1. Identification of topic (building relationships or developing research idea)	49 (69%)
	2. Design or development	45 (63%)
	3. Conduct	35 (49%)
	4. Analyses	10 (14%)
	5. Dissemination	28 (39%)
	Participation in at least one of the two formative stages (stages 1 or 2)	62 (87%)
	Participation in all 5 stages	8 (11%)
Mode of participation	Consultative	35 (49%)
	Collaborative	29 (41%)
	Adolescent-led	6 (9%)
	Other^*^	1 (1%)
Participatory outcomes (PO) measured	Sense of self-worth or self-esteem or efficacy	5 (7%)
	Being taken seriously	2 (3%)
	Making decisions	2 (3%)
	Public civic engagement	0%
	No. of studies which measured at least one PO	5 (7%)
	No. of studies which measured all four PO	1 (1%)
Chronic disease related outcomes (CDO)^**^	Increased Awareness	49 (69%)
	Program or intervention development	45 (63%)
	Policy change	8 (11%)
	Environmental change	16 (23%)
	Behavior change (diet and physical activity)	18 (25%)
	Health status or risk factors change^***^ (wt. = 6, BP = 1)	7 (10%)
	No. of studies which reported four or more CDO	10 (16%)
Models, theories or conceptual frameworks n (%)	Participatory^****^	34/71 (48%)
	Social^*****^	34/71 (48%)
	Complex models or multiple theories	35/71 (49%)
	Other^******^	21/71 (30%)
	Not reported	9/71 (13%)

* *Adolescents played a key role as facilitators, however they did not play any part in decision-making or influencing the process*.

** *These were not always reported or aimed for*.

*** *Six studies reported change in weight, one study reported a change in blood pressure*.

**** *Participatory alone (13), participatory included in multi-theory (21)*.

***** *Social theory alone (5), Socio-ecological theory (7), Socio-cognitive theory (22, 31%)*.

****** *Behavioral (3), Ecological (8), Environmental (1), Psychological (2), Empowerment (7)*.

#### Participatory Methods

The number and type of participatory methods used by researchers in the included studies significantly varied. Results revealed that a combination of methods was commonly employed in each study. Approaches ranged from one participatory method per study to a maximum of four methods, with a mean of two participatory methods per study. Adolescent-led participatory approaches were more likely to employ a greater number of participatory methods (mean of three methods per study) to achieve their outcomes.

##### Components of Participatory Methods

Overall, the most common single method used was focus groups in 55% (39/71) of studies, followed by questionnaires and surveys (38%, 27/71) and interviews (38%, 27/71). These were more commonly employed in consultative studies either alone or combination with other methods. Furthermore, other methods such as photovoice (*n* = 10), needs and community assessments (*n* = 5), discussion groups, and community forums (*n* = 3) were also used in a smaller proportion of studies. These methods have in common that they elicit views, opinions and uncover the needs of adolescents and their community which is a fundamental element of formative work. Youth advisory groups (*n* = 2) and peer leadership and advocacy activities were a component of 25% of studies (18/71). The studies that used these approaches were typically studies that employed a collaborative (*n* = 13) or adolescent-led (*n* = 3) mode of participation. A social marketing approach was used in six studies (8%), four of which were collaborative, and two were adolescent-led. Additionally, co-design or co-creation activities (sessions) were specified in 7% (*n* = 5) of studies; these were a component of one consultative study and four studies that used a collaborative approach.

### Models, Theories, and Conceptual Frameworks

Overall, studies varied significantly in their theoretical basis, with 9 theoretical models and 15 complex models and frameworks reported. Table 3 presents the most common models, theories and frameworks used. A significant number of studies used multiple theories or models (49%, 35/71). Overall, 48% (34/71) of studies reported a participatory design, of which 74% (25/34) used CBPR principles to guide their project. Moreover, other theories which complement but do not explicitly promote a participatory approach were identified. Thirty-four (48%) studies reported a social component, with social-cognitive theory reported to inform 22/71 (31%) of studies, and socio-ecological theories informed seven studies. Of the multi-theory models employed, which did specifically involve a participatory component, the transtheoretical model of behavior change (*n* = 5) and the PRECEDE-PROCEED model (*n* = 4) were the most reported. Empowerment theory was used in seven studies; however, participatory outcomes which measure empowerment and influence were only measured in five studies. Of the studies labeled participatory, 32% (11/34) involved adolescents in at least four research cycle stages. A collaborative approach was employed in more than half (18/34, 53%) of participatory studies.

### Chronic Disease Outcomes

Chronic disease outcomes were largely based on outcomes of obesity prevention research. The most common chronic disease-related outcomes of adolescent participation were “increased awareness among family and peers” (49/71, 69%) and “program or intervention development” (45/71, 63%). It is also important to note that two studies (69, 135) were in progress, and therefore, information on outcomes was not available or incomplete.

#### Characteristics of Studies Reporting Four or More Chronic Disease Outcomes

Overall, 12 studies (17%, 12/71) reported four or more chronic disease outcomes; these studies tended to be 12 months or longer in duration, and five of these studies (41%) involved adolescents in four or more stages of the research cycle. Nine of the 12 studies (75%) used a mixed-methods interventional design, with seven studies (58%) involving an RCT component and another two (17%) studies using a quasi-experimental design. Of these 12 studies, 11 (92%) employed a collaborative mode of adolescent participation. Moreover, seven studies (58%) were supported by multiple theoretical frameworks, and six (50%) had a participatory agenda. While 8/12 (67%) studies used youth or peer leadership or advocacy practice, only 33% (4/12) were also described as participatory. In contrast, all seven RCTs involved youth or peer-leadership or advocacy regardless of whether they declared the usage of a participatory model or not. Overall, participatory outcomes were measured in 5/71 (7%) studies but were more likely to be reported in studies with four or more chronic disease outcomes measured 3/12 (25%).

#### Characteristics of Studies Reporting Three or Fewer Chronic Disease Outcomes

Due to the broad range of study designs and aims, chronic disease outcomes as per the established categories were not within the aim or scope of many of the studies reviewed and, as such, were not reported. This was particularly the case in qualitative studies that employed a consultative participatory mode, usually focus groups, discussion groups, questionnaires, surveys, or interviews. In these instances, the researchers were seeking opinions and preferences. Therefore changes in weight, diet, or physical activity levels were not measured as they would be outside the scope of the study. A total of 91% (29/32) of consultative studies described <3 chronic disease outcomes. Similarly, all six studies (82, 86, 114, 133, 173) that used an adolescent-led participation mode achieved no more than three chronic disease outcomes despite all except one (145) meeting the aims of their respective projects. Furthermore, adolescent-led studies described outcomes related to increased awareness (82, 86, 133, 173), program/intervention development (86, 114, 133, 173), or environmental changes (86, 133) and did not report anthropometric, behavioral changes, or changes in health status associated with obesity and chronic disease development.

### Effectiveness of Studies Involving Adolescent Participation in Improving Obesity and Chronic Disease Risk Factors

Differences in the study designs and methodologies of the studies analyzed meant that data reporting differed. Quantitative measures were often not measured/ or sought in many studies included within this review. Still, 22 studies (31%) reported an improvement in one or more obesity risk factors. Data on anthropometric measures as well as behavioral measures such as physical activity and dietary intake were analyzed.

#### Anthropometric Measures

Eight studies (11%) reported an improvement in one or more anthropometric measures such as BMI (57, 62, 76, 91, 158, 169), weight (161), waist circumference (158), or adiposity (142) albeit not always statistically significant (161, 169). Methodological components which were common between these studies included use of an RCT (57, 62, 76, 91, 142, 169) in 75% (6/8) of studies, a collaborative (62, 76, 91, 158, 161, 169) mode of adolescent participation in 75% (6/8) of studies and inclusion of a peer-component (57, 62, 76, 91, 169) in 63% (5/8) of studies.

#### Physical Activity and Fitness

Improvements in physical activity and fitness behaviors were reported in 12 studies (54, 93, 96, 101, 105, 126, 142, 149, 161, 164, 165, 169), five of these involved an RCT (101, 142, 164, 165, 169), seven employed a collaborative (54, 93, 96, 105, 161, 165, 169) mode of adolescent participation and seven involved a peer-component (93, 96, 105, 126, 149, 164, 169).

#### Dietary Behaviors

Encouraging changes in dietary behaviors were reported in 17 studies, as an improvement in dietary intake or nutrition behaviors (61, 91, 96, 101, 105, 112, 126, 149, 161, 164, 165, 169), increased fruit (93) and vegetable consumption (121), reduced caloric intake (99), reduction in sugar sweetened beverage intake (108) or an increase in water consumption (54). Overall studies involving improvements in dietary behaviors had in common that they were more likely to be collaborative (9/17) (54, 91, 93, 96, 105, 108, 161, 165, 169) and involve a peer-component (10/17) (61, 91, 93, 96, 105, 108, 126, 149, 164, 169). Studies which reported an improvement in dietary behaviors were almost evenly of qualitative (7/17) (54, 93, 105, 112, 121, 126, 149) or RCT (6/17) design (91, 101, 108, 164, 165, 169).

### Barriers and Facilitators to Adolescent Engagement

Understanding barriers and facilitators to adolescent engagement are essential in improving meaningful adolescent participation in the obesity prevention research cycle. Of the studies analyzed, school, family, and work commitments were the most common barriers suggested. Facilitators to participation included compensation for the time spent participating, peer-support, leaderships opportunities, and adolescent-led participatory methods (such as photovoice and youth advisory groups), which gave the youth a voice and influence. Furthermore, incorporating meaningful participation into supportive and trusted environments such as the local community or school setting was suggested to facilitate engagement.

Overall, barriers and facilitators to adolescent participation when documented were usually reported as general observations rather than concrete data. Barriers and facilitators to participation were seldom an outcome measured in the studies reviewed and is in line with lacking measurements of participatory outcomes in general.

## Discussion

This is the first review investigating adolescent participation in the obesity prevention literature to the best of our knowledge. Overall, findings from our review indicate that adolescent participation in research is a complex yet adaptable construct that may be a vital link between obesity prevention research and practice.

Our review detailed the new and emerging field of adolescent participatory research in obesity prevention, with more than 50% of the studies identified having published their first paper in the last 5 years. We found that despite obesity and its associated co-morbidities affecting populations broadly (176), the use of participatory research methods were not equitably deployed. Of the studies which reported socioeconomic characteristics, we found that 85% targeted minority or underserved communities in mostly affluent western nations. Only four studies took place in middle-income nations, where adolescents represent a greater proportion of the total population (177) and where obesity has surpassed undernutrition as a leading cause of morbidity (4). The lack of participatory research for adolescents in LMICs may be attributable to low resources and challenges in health research allocation and implementation. Funders and the global community often expect LMICs to direct research and funds to the most acute public health challenges (178). Furthermore, our finding suggests adolescent participation in obesity prevention research appears to be gender-biased with studies involving predominantly adolescents identifying as female. Adolescents identifying as male were either not represented at all or underrepresented in 55% of the included studies, while “no representation” or underrepresentation of adolescents identifying as female-only occurred in only 11% of studies. This finding may be explained by sociocultural factors and gender-based ideals, particularly in higher-income countries, with females more likely to be concerned with weight compared to their male counterparts (2). Girls are also more likely to partake in disordered eating behaviors and calls for further public health attention (179).

Our review revealed that over the last decade there has been a gradual increase in the awareness of the need to involve adolescents in the dialogue and decision-making processes to make progress on matters affecting youth, such as the global obesity crisis. This finding is consistent with the WHO-UNICEF-*Lancet* commission report (180), which recommends that youth be placed at the center of Sustainable Development Goals (SDGs). The commission states that “children should be given high-level platforms to share their concerns and ideas and to claim their rights to a healthy future” (180). Insights from our review indicate that meaningful participation not only upholds adolescents' fundamental right to participation but also allows for the development of interventions that are tailored to the unique needs of this demographic.

Results of our review suggest that meaningful adolescent participation is limited within obesity and chronic disease prevention research. Our results indicate that when adolescents are engaged, they are more likely to be involved in the formative stages of the research cycle in activities such as relationship building, needs assessment, research idea development, and project design (34). Nevertheless, only 11% of the included studies reported meaningful participation in all stages of the research cycle. Further, our review determined that to date, participatory research with young people has been predominantly consultative in nature, using methods such as surveys, focus groups, and interviews to elicit views and opinions. At the same time, an adolescent-led mode of participation was described in only 9% of the 71 studies examined. We found that methods that engage adolescents more meaningfully in research such as YAGs, youth advocacy, and peer leadership were a common component of the few adolescent-led participatory studies. Furthermore, studies that involved a youth-led component reported improvements in capacity building markers of empowerment and influence, enhanced confidence, and gave adolescents a voice in research that concerns their lives. The use of YAGs exemplifies a novel (181) yet practical and effective strategy to meaningfully engage adolescents in an adolescent-led approach throughout the entirety of the research cycle (114, 130). Similarly, in a recent scoping review investigating the use of YAGs in health research, Sellars et al. determined that YAGs are a valuable yet underutilized method of involving young people in effective research development and translation globally (34). Moreover, Sellars et al. also found an underutilization of YAGs was more apparent in research conducted in low and middle-income countries (LMICs), where socioeconomic and cultural factors such as lack of resources and age discrimination limited the use of such engagement strategies (34). This finding is consistent with the findings of our review, which found few participatory-focused research studies for adolescent obesity prevention in LMICs.

Findings from our review indicate significant variability in the scope of reported outcomes of meaningful adolescent participation. We found that participatory outcomes are rarely evaluated, and chronic disease outcomes are inconsistently reported. Nevertheless, similarly to Larsson et al. (38), our review provided evidence that increasing adolescent involvement in the advisory, co-design, and decision-making processes contributed to more meaningful obesity and chronic disease prevention associated outcomes. Chronic disease outcomes described within the studies analyzed included increased awareness of obesity, its risk factors and prevention, obesity prevention interventions and program development, environmental modifications to promote healthier lifestyle habits, behavioral changes including diet and physical activity, and improvements in anthropometric measures such as BMI and weight. Furthermore, we identified that meaningful participation contributes to the development of research and leadership skills as well participatory outcomes of empowerment and influence (82, 86). Although participatory outcomes were rarely evaluated in the 71 studies included within this review, there were reports of improved outcomes for the participating youth (97) and their peers (57, 62, 96). Additionally, DeBar et al. reported in their multi-center RCT, that meaningful participation alone (regardless of the amount) was the main factor driving a positive effect on outcomes, with peer-leadership and commitment contributing to a significant improvement in dietary behavior outcomes as well as a reduction in BMI compared to controls (76).

Our review revealed that current research is failing to take into consideration the dynamic context in which adolescents live, participate and the associated social, economic, cultural, political and health policy influences (182). Most included studies in our scoping review (87%) involved adolescent participation within a single socio-ecological setting, namely, local communities and secondary education institutions. However, studies that also included a “peer” context component in the form of YAGs, leadership, advocacy, or peer education were found to be more likely to achieve a wider range of positive chronic disease outcomes. Moreover, findings from our review reinforced that adolescents as individuals live within a complex and intertwined socio-ecological sphere, which often impacts their opportunity for health equity (6, 25). In each context, adolescents experienced barriers and facilitators to their diet and lifestyle choices, and decision-making (183). Engaging adolescents in meaningful obesity prevention research across socio-ecological domains may allow for more efficient identification of risk factors that contribute to global adolescent health inequalities resulting in more dynamic intervention development and implementation (184–186). A study by Livingood et al. provided an exemplar model of how to engage adolescents in meaningful participation in research (114). Youth were engaged in a multi-method adolescent-led approach throughout the entirety of the research cycle. Youth developed and facilitated a youth advisory board and used methods such as photovoice and focus groups to identify needs, concerns and preferences. Youth analyzed and presented their findings, which ultimately led to the development of a digital communication (mHealth) obesity prevention intervention that was tailored to the specific needs of youth. However, although obesity prevention was indicated as an objective of the aforementioned study, chronic disease-related outcomes such as anthropometric measures were not taken and hence it is unclear what impact the development of this youth-led intervention will have on obesity incidence within the community (114).

### Limitations

Despite our best efforts, this scoping review has several limitations and challenges to note. Overall, it was recognized that due to the lack of standardized measures and reporting, measuring and documenting participatory and chronic disease outcomes were predominantly subjective in nature. Furthermore, the term “participation” is routinely used to refer to participants taking part in a study and not necessarily participating in a meaningful way as per participatory frameworks (25). This made it difficult to decipher if meaningful participation was a component of the research processes. Additionally, participatory approaches were often included in methodology sections of reported studies and hence were often not components of the outcomes measured.

Furthermore, scoping reviews have innate limitations of importance to consider. By design scoping reviews are broad in scope and aim to map the literature therefore, the included studies were heterogenous in range of study methodologies and designs. This made direct comparisons between studies challenging. Furthermore, although search terms used were broad and the search strategy was systematic, as with any review, it is possible that some studies were missed. Moreover, studies were limited to those with abstracts published in English; this self-selection limitation may have inadvertently excluded perspectives from non-English speaking countries. Finally, as per the scoping review guidelines, it is not necessary to rate the quality of the data or conduct a critical appraisal of the evidence used in scoping reviews; this may have implications for practice (47).

### Recommendations

For research to translate effectively into practice and to address health equity challenges faced by minority and underserved communities, more effort needs to be placed on making participatory research gender-balanced and inclusive. Future research should endeavor to identify why high-risk groups such as males in high and high-middle-income countries are underrepresented in obesity prevention participatory research and work toward facilitating equal representation between genders. Furthermore, future participatory obesity prevention research should aim for a broad representation of young people from different socio-economic backgrounds as well as cultural and social groups. Additionally, funding bodies and researchers should direct attention and resources to the growing adolescent obesity concerns in LMICs, where meaningful adolescent participation in obesity prevention research is remarkably lacking. Also, for participatory research to truly capture the voice and needs of adolescents, it is necessary for researchers to engage young people in an increasingly collaborative or adolescent-led capacity throughout all stages of research and development processes. Researchers should also take into consideration the diverse and dynamic socio-ecological settings in which adolescents and their peers connect. Finally, to adequately measure participatory outcomes and make comparisons between studies we recommend standardized and universal tools to measure participatory outcomes in adolescent obesity-related participatory research.

## Conclusion

Findings from our review indicate that adolescents globally are not being engaged sufficiently in obesity prevention research decision-making to uphold the recommendations of the WHO-UNICEF-Lancet commission. The limited number of studies identified from this review that engaged adolescents to a greater capacity within the research process highlights a key opportunity for enhancing obesity prevention research and practice. Meaningful engagement of adolescents in an inclusive and fair manner builds their capacity to contribute throughout the obesity prevention research process. Addressing the unique needs of adolescents requires adolescents to be afforded increased opportunities to collaborate and lead stages of the decision-making, research and translation process.

## Author Contributions

MM, SM, HC, PP, JR, and SP: conceptualization, methodology, investigation, and writing—review and editing. MM: writing—original draft preparation. JR and SP: supervision. All authors have read and agreed to the published version of the manuscript.

## Funding

This research was supported by a National Health and Medical Research Council/National Heart Foundation Early Career Fellowship (Grant Number APP1157438) awarded to SP. While JR was funded by a National Health and Medical Research Council Career Development Fellowship (Grant Number APP1143538).

## Conflict of Interest

The authors declare that the research was conducted in the absence of any commercial or financial relationships that could be construed as a potential conflict of interest.

## Publisher's Note

All claims expressed in this article are solely those of the authors and do not necessarily represent those of their affiliated organizations, or those of the publisher, the editors and the reviewers. Any product that may be evaluated in this article, or claim that may be made by its manufacturer, is not guaranteed or endorsed by the publisher.

## Supplementary Material

Supplementary File 1 Prisma Scoping Review Checklist.

Supplementary Table 1 Medline Search Strategy.

Supplementary Table 2 Data extraction table of included studies.

## Abbreviations

USA: United States of America
UNICEF: United Nations Children's Fund
NIHR: National Institute for Health Research
NHMRC: National Health and Medical Research Council
PAR: Participatory Action Research
PE: Patient Engagement
CBPR: Community Based Participatory Research
PRISMA-ScR: Preferred Reporting Items for Systematic Reviews and Meta-Analyses extension for Scoping Reviews
MeSH: Medical Subject Headings
CINAHL: Cumulative Index of Nursing and Allied Health Literature
CENTRAL: Cochrane Controlled Register of Trials
RCT: Randomized Control Trial
SES: Socioeconomic Status
BMI: Body Mass Index
WHO: World Health Organization
SDGs: Sustainable Development Goals
YAG: Youth Advisory Group
LMICs: Low- and Middle-Income Countries.
