# Supplementary material for: Shifting From Tokenism to Meaningful Adolescent Participation in Research for Obesity Prevention: A Systematic Scoping Review
Source: Front Public Health. 2021 Dec 23;9:789535. doi: 10.3389/fpubh.2021.789535 (PMC8734426; doi:10.3389/fpubh.2021.789535)
Supplement: Supplementary Table 1 — Medline Search Strategy. [file Table_1.docx]

Supplementary Table 1: Medline Search Strategy (17.02.2020)

| **#** | **Searches** | **Results** |
| --- | --- | --- |
| 1 | (nutrition* or diet* or “physical activity” or health*).mp. [MP=title, abstract, original title, name of substance word, subject heading word, floating sub-heading word, keyword heading word, organism supplementary concept word, protocol supplementary concept word, rare disease supplementary concept word, unique identifier, synonyms] | 4393981 |
| 2 | ((“over weight” or obesity or “pediatric obesity” or “paediatric obesity” or “lifestyle risk factors” or “non-communicable diseases” or “noncommunicable disease”) and prevention).mp. [mp=title, abstract, original title, name of substance word, subject heading word, floating sub-heading word, keyword heading word, organism supplementary concept word, protocol supplementary concept word, rare disease supplementary concept word, unique identifier, synonyms] | 49261 |
| 3 | (youth or adolescen* or teen*).mp. [mp=title, abstract, original title, name of substance word, subject heading word, floating sub-heading word, keyword heading word, organism supplementary concept word, protocol supplementary concept word, unique identifier, synonyms] | 2087732 |
| 4 | (participat* or involvement or engagement or “co-design” or “co design” or “decision making” or “decision-making”.mp. [mp=title, abstract, original title, name of substance word, subject heading word, floating sub-heading word, keyword heading word, organism supplementary concept word, protocol supplementary concept word, protocol supplementary concept word, rare disease supplementary concept word, unique identifier, synonyms] | 1195048 |
| 5 | (research or guidelines or policy or policies or “action research”).mp. [mp=title, abstract, original title, name of substance word, subject heading word, floating sub-heading word, keyword heading word, organism supplementary concept word, protocol supplementary concept word, protocol supplementary concept word, rare disease supplementary concept word, unique identifier, synonyms] | 10639503 |
| 6 | 1 and 2 and 3 and 4 and 5 | 999 |
| 7 | Exp Humans/ | 18302997 |
| 8 | 6 and 7 | 962 |
| 9 | Health Promotion/ or Community-Based Participatory research/ or Health Services Research/ or participatory .mp. or Health Knowledge, Attitudes, Practice/ | 220050 |
| 10 | 8 and 9 | 443 |
| 11 | 10 and 1995:2020.(sa_year). | 438 |
