# Supplementary material for: Shifting From Tokenism to Meaningful Adolescent Participation in Research for Obesity Prevention: A Systematic Scoping Review
Source: Front Public Health. 2021 Dec 23;9:789535. doi: 10.3389/fpubh.2021.789535 (PMC8734426; doi:10.3389/fpubh.2021.789535)
Supplement: Supplementary Table 2 — Data extraction table of included studies. [file Table_2.docx]

Supplementary Table 2: Data extraction table of included studies

|  |  |  |  | **Adolescent Participation in the Research Cycle** | | | | | **Adolescent engagement: Mode of participation** | | | |  | **Participatory Outcomes measured**  ***(characterised by empowerment & influence)-*** | | | | **Chronic Disease Related Outcomes** | | | | | |
| --- | --- | --- | --- | --- | --- | --- | --- | --- | --- | --- | --- | --- | --- | --- | --- | --- | --- | --- | --- | --- | --- | --- | --- |
| **Reference** | **Study Design/ Methods** | **Main Chronic disease or risk factor** | **Socio-ecological Setting** | **1. Identification of topic** | **2. Design & Development** | **3. Conduct** | **4. Analyses** | **5. Dissemination** | **Consultative** | **Collaborative** | **Adolescent-led** | **Other:** Adolescents engaged but views did not influence design or development of the intervention | **Model/ theory/ conceptual framework** | **Sense of self-worth/ self-esteem/ efficacy** | **Being taken seriously** | **Making decisions** | **Public/civic engagement** | **Increased awareness** (among family, or peers, community groups, etc) | **Program/ intervention development** | **Policy change** | **Environmental change** (facilities, safe spaces, accessibility to healthier food choices) | **Behaviour change** (Diet/ physical activity) | **Health status/risk factors change** (e.g., weight change, BP change, etc) |
| Anselma et al, 2018 Netherlands (1)  Anselma et al, 2019 Netherlands (2)  Anselma et al, 2019 Netherlands (3)  Anselma et al, 2020 Netherlands (4) | **Qualitative**  Needs assessment Focus groups  Intervention Mapping (IM) | Overweight/ obesity | Local community |  |  |  |  |  |  | Y |  |  | Multiple model (incl. participatory- YPAR)) |  |  |  |  |  |  |  |  | Physical activity water policy |  |
| Arlinghaus et al, 2017, USA (5) | **RCT**  Adolescent health mentors/ leaders | Obesity | Institution (secondary education) |  |  |  |  |  |  |  |  | Y | NR | NR | NR | NR | NR |  |  |  |  |  |  |
| Bailey et al, 2018, Australia (6) | **Qualitative**  Focus groups | Overweight/  Obesity | Local community |  |  |  |  |  | Y |  |  |  | Participatory | NR | NR | NR | NR |  |  |  |  |  |  |
| Balvanz et al, 2016, USA (7) | **Qualitative**  Photovoice- Youth advocacy | Obesity | Local community |  |  |  |  |  |  | Y |  |  | Multiple model (incl. CBPR) | NR | NR | NR | NR |  |  |  |  |  |  |
| Bardwell et al, 2009, USA (8) | **Qualitative**  Questionnaire  Participatory activities | Obesity | Family & Local community |  |  |  |  |  |  | Y |  |  | Participatory (CBPR) | NR | NR | NR | NR |  |  |  |  |  |  |
| Uyeda et al, 2009, USA (9)  Goh et al, 2009, USA (10)  Bogart et al, 2011, USA (11)  Bogart et al, 2014, USA (12)  Bogart et al, 2016, USA (13)  Bogart et al, 2018, USA (14) | **Mixed methods + RCT**  Focus groups/ Questionnaires/Peer leadership/ Peer advocacy/  Marketing | Obesity | Institution (secondary education) |  |  |  |  |  |  | Y |  |  | Multiple theory incl. participatory (CBPR), SCT | NR | NR | NR | NR |  |  |  |  |  |  |
| Christianson, et al. 2019, USA (15) | **Qualitative**  Social marketing  Focus groups | Wellness | Institution (tertiary education) |  |  |  |  |  |  | Y |  |  | Multiple theory (incl. participatory (CBPR)) | NR | NR | NR | NR |  |  |  |  |  |  |
| Chae, et al, 2016, Republic of Korea (16) | **Qualitative**  Focus groups | Overweight/obesity | Institution (secondary education) |  |  |  |  |  | Y |  |  |  | Ecological model | NR | NR | NR | NR |  |  |  |  |  |  |
| Cassidy et al, 2013, USA (17) | **Mixed** methods  Focus groups Questionnaires Role plays | Overweight/ obesity/ | Local community |  |  |  |  |  | Y |  |  |  | Multiple theory (incl. participatory (CBPR)) | NR | NR | NR | NR |  |  |  |  |  |  |
| Brown et al, 2010, USA (18)  Brown et al, 2013, USA (19) | **Qualitative**  Focus groups/ Interviews | Diabetes | Local community |  |  |  |  |  | Y |  |  |  | Multiple theory (incl. participatory (CBPR), SGT) | NR | NR | NR | NR |  |  |  |  |  |  |
| Diez-Canseco, 2015, Peru (20) | **Qualitative**  Peer promotion (education, training & feedback)  Focus groups | Non- communicable diseases.  (NCD) | Institution (Secondary education) |  |  |  |  |  |  | Y |  |  | Participatory | NR | NR | NR | NR |  |  |  |  |  |  |
| Correa et al, 2017, Canada (21)  Rajaraman et al, 2015, Canada (22) | **Qualitative**  Focus groups | Overweight/ obesity | Adolescent (Individual) |  |  |  |  |  | Y |  |  |  | Multiple/complex theory | NR | NR | NR | NR |  |  |  |  |  |  |
| Cueva et al, 2019, USA (23) | **Qualitative**  Photovoice  Discussions | Obesity | Local community |  |  |  |  |  | Y |  |  |  | Multiple theory incl. participatory (CBPR) | NR | NR | NR | NR |  |  |  |  |  |  |
| DeBar et al, 2011, USA (24)  DeBar et al, 2009, USA (25)  HEALTHY study group, 2005,2009, USA (26, 27)  Siega-Riz et al,, 2011, USA (28)  Venditti, et al 2005, 2009, USA (29, 30)  Marcus et al, 2013, USA (31) | **RCT**  Focus groups  Peer leadership  Public commitment activities  Social marketing | Obesity, Diabetes | Institution  (secondary education) |  |  |  |  |  |  | Y |  |  | NR | NR | NR | NR | NR |  |  |  |  |  |  |
| de Vet, et al, 2013, Netherlands (32) | **Qualitative**  Cross-sectional survey | Overweight/ obesity | Institution (secondary education) |  |  |  |  |  | Y |  |  |  | NR | NR | NR | NR | NR |  |  |  |  |  |  |
| Edwardson et al, 2015, UK (33)  Harrington et al, 2018, UK (34) | **RCT**  Questionnaires  Peer tutoring peer modelling peer leadership marketing  Focus groups | Poor physical activity | Institutions (secondary education) |  |  |  |  |  |  | Y |  |  | Social, Cognitive Theory | NR | NR | NR | NR |  |  |  |  |  |  |
| Ewan et al, 2015, USA (35) | **Qualitative** Concept mapping. | Obesity | Institution/ local community |  |  |  |  |  |  | Y |  |  | Multi-model  (Participatory &  Social ecological model of health) | NR | NR | NR | NR |  |  |  |  |  |  |
| Findholt, et al, 2010, USA (36) | **Qualitative**  Photovoice | Obesity | Local community |  |  |  |  |  |  |  | Y |  | Participatory (CBPR) | NR | NR | NR | NR |  |  |  |  |  |  |
| Frerichs et al, 2012, USA (37)  Frerichs et al, 2015, USA (38) | **Qualitative**  Interviews  Social marketing (including youth generated branding) & Social Media,  Youth advocacy | Obesity | Local community |  |  |  |  |  |  |  | Y |  | Multi-model (incl. SCT) | NR | NR | NR | NR |  |  |  |  |  |  |
| Frerichs et al, 2018, USA (39) | **Qualitative**  Surveys Discussions-  Testing systems science curriculum | Obesity | Local community |  |  |  |  |  | Y |  |  |  | Multi-model (incl.  Participatory  (CBPR) and SCT) | NR | NR | NR | NR |  |  |  |  |  |  |
| Garcia et al 2019, USA (40) | **Qualitative**  Focus groups  Short survey | Obesity | Local community |  |  |  |  |  | Y |  |  |  | Multi /complex-model (PRECEDE-PROCEED planning model & Socio-ecological model) | NR | NR | NR | NR |  |  |  |  |  |  |
| Garnett et al, 2015, USA (41) | **Qualitative**  Community forum (informal facilitated group discussion) | Over-weight/ Obesity | Local community |  |  |  |  |  | Y |  |  |  | Multi-model incl.  Participatory (CBPR Conceptual Logic Model (Wallerstein & Duran))  c | NR | NR | NR | NR |  |  |  |  |  |  |
| Green et al, 2019, USA (42) | **Qualitative**  Youth leadership  Questionnaires/ Surveys.  Health campaign | Obesity | Local community |  |  |  |  |  |  | Y |  |  | Multi-theory  (incl. Participatory- CBPR, Socio-ecological theory +SCT) | NR | NR | NR | NR |  |  |  |  |  |  |
| Hagedorn et al, 2018, USA (43) | **MIXED methods**  Teen leaders/ educators/ student researchers  Discussion | Obesity | Local community |  |  |  |  |  |  | Y |  |  | Social Learning Theory Framework | NR | NR | NR | NR |  |  |  |  |  |  |
| Grignard et al, 2003, Belgium (44) | **MIXED methods**  Questionnaire, body size dissatisfaction test & interview | Obesity | Adolescent (individual) |  |  |  |  |  | Y |  |  |  | NR | NR | NR | NR | NR |  |  |  |  |  |  |
| Hannay et al, 2013, USA (45) | **Qualitative**  Photovoice workshops, focus groups  Advocacy | Obesity | Local community |  |  |  |  |  |  | Y |  |  | Participatory- CBPR | NR | NR | NR | NR |  |  | ? |  |  |  |
| Jackson et al, 2010, USA (46) | **Qualitative**  Focus groups Development of a theatre- based intervention | Overweight | Local community |  |  |  |  |  |  | Y |  |  | Participatory-CBPR | NR | NR | NR | NR |  |  |  |  |  |  |
| Hunsberger et al, 2014, USA (47) | **Mixed** methods  Interviews | Over-weight/ obesity | Institution  (Secondary education) |  |  |  |  |  | Y |  |  |  | Participatory-CBPR | NR | NR | NR | NR |  |  |  |  |  |  |
| Knoblock-Hahn et al, 2016, USA (48)  Le Rouge et al, 2019 USA (49)  Le Rouge et al, 2020, USA (50) | **Qualitative**  Focus groups  Design  Usability sessions | Over-weight/ obesity | Adolescent (individual) |  |  |  |  |  | Y |  |  |  | Multi/complex model (incl. SCT) | NR | NR | NR | NR |  |  |  |  |  |  |
| Kong et al, 2011, USA (51)  Sussman et al, 2013, USA (52) | **MIXED**  Interviews and surveys  Community Advisory Council (unsure how many were youth) | Obesity | Institution  (secondary education) |  |  |  |  |  |  | Y |  |  | Multi-model (incl. Participatory- CBPR + socio-ecological) | NR | NR | NR | NR |  |  |  |  |  |  |
| Kramer et al, 2010, USA (53)  Kramer et al, 2012, USA (54) | **Qualitative**  Photovoice  Advocacy  Presentation of findings | Obesity | Local community |  |  |  |  |  |  | Y |  |  | Participatory-CBPR | NR | NR | NR | NR |  |  |  |  |  |  |
| Lane et al, 2018, USA (55) | **Qualitative**  Surveys | Obesity | Institution  (secondary education) |  |  |  |  |  | Y |  |  |  | NR | NR | NR | NR | NR |  |  |  |  |  |  |
| Lewis et al, 2018, USA (56) | **Qualitative**  Surveys  Helped plan summer enrichment camp activities | Obesity | Institution  (secondary education) |  |  |  |  |  | Y |  |  |  | Participatory-CBPR | NR | NR | NR | NR |  |  |  |  |  |  |
| Linton et al, 2014, USA (57)  Milstein et al, 2016, USA (58) | **Qualitative**  Surveys  Community assessment  Youth advocacy | Obesity | Local community |  |  |  |  |  |  | Y |  |  | Multi-theory (incl. SCT) |  |  |  | NR |  |  |  |  |  |  |
| Livingood et al, 2017, USA (59) | **Qualitative** Youth advisory board  Photovoice Focus group Interviews | Obesity | Local community |  |  |  |  |  |  |  | Y |  | Multi-theory (incl. participatory-CBPR, eco model) | NR | NR | NR | NR |  |  |  |  |  |  |
| Llaurado et al, 2015, Spain (60)  Tarro et al, 2017, Spain (61)  Aceves-Martins, 2017, Spain (62)  Tarro et al, 2019, Spain (63)  Llaurado et al, 2018, Spain (64) | **RCT**  Peer-led  Focus groups  Social marketing | Obesity | Institution  (secondary education) |  |  |  |  |  |  | Y |  |  | Multi-model (incl. social) | NR | NR | NR | NR |  |  |  |  |  |  |
| Loman, 2008, USA (65) | **Qualitative**  Focus groups and interviews | Overweight/ obesity | Local community |  |  |  |  |  | Y |  |  |  | SCT | NR | NR | NR | NR |  |  |  |  |  |  |
| Mauriello et al, 2006, USA (66)  Mauriello et al, 2010, USA (67) | **Qualitative**  Surveys  Focus groups, Interviews | Obesity | Institution (secondary education) |  |  |  |  |  | Y |  |  |  | Complex model (Transtheoretical Model framework) | NR | NR | NR | NR |  |  |  |  |  |  |
| Maynard et al, 2009, UK (68)  Rawlins et al, 2013, UK (69)  Maynard et al, 2017, UK (70) | **MIXED**  Focus groups Interviews Questionnaires | Obesity | Local community |  |  |  |  |  | Y |  |  |  | Multi/complex models | NR | NR | NR | NR |  |  |  |  |  |  |
| McGee et al, 2017, USA (71) | **Qualitative**  Focus groups | Obesity | Local community |  |  |  |  |  | Y |  |  |  | The Social Cognitive Theory (SCT) | NR | NR | NR | NR |  |  |  |  |  |  |
| McKinney et al, 2014, USA (72) | **Qualitative**  Peer educators  Surveys  Focus groups | Obesity | Institution (secondary education) |  |  |  |  |  | Y |  |  |  | Multi- model (incl. Participatory-CBPR + SCT) | NR | NR | NR | NR |  |  |  |  |  |  |
| McPherson, and Lindsay, 2012, Canada (73) | **Qualitative**  Focus groups | Obesity | Institution (hospital) |  |  |  |  |  | Y |  |  | Y | NR | NR | NR | NR | NR |  |  |  |  |  |  |
| Morales-Campos, et al, 2015, USA (74) | **Qualitative**  Community advisory board partners  Surveys  Intervention planning  Photo mapping | Obesity | Local community |  |  |  |  |  |  | Y |  |  | Multi theory (incl. Participatory-CBPR + SCT) | NR | NR | NR | NR |  |  |  |  |  |  |
| Mosavel, et al, 2018, USA (75) | **Qualitative**  Asset mapping (part of a needs assessment) | Obesity | Local community |  |  |  |  |  |  | Y |  |  | Participatory- CBPR | NR | NR | NR | NR |  |  |  |  |  |  |
| Muturi et al, 2018, USA (76) | **Qualitative**  Survey | Obesity | Adolescent (Individual) |  |  |  |  |  | Y |  |  |  | Multi-theory (incl. participatory) | NR | NR | NR | NR |  |  |  |  |  |  |
| Necheles et al, 2007, USA (77) | **Qualitative**  Photovoice  Interviews  Social marketing  Youth advocacy | Health issues, health behaviours, unhealthy food, obesity | Local community |  |  |  |  |  |  |  | Y |  | Multi-theory (incl. participatory- CBPR) | NR | NR | NR | NR |  |  |  |  |  |  |
| Nguyen et al, 2017, Vietnam (78)  Tang et al, 2020, Vietnam (79) | **Qualitative,**  Planned Pilot- RCT  Peer education, Peer support, Focus groups, Interviews | Obesity | Institution (secondary education) |  |  |  |  |  | Y |  |  |  | Multi-theory  (incl. SCT) | NR | NR | NR | NR |  |  |  |  |  |  |
| Partridge et al, 2019, Australia (80)  Partridge et al, 2020, Australia (81) | M**ixed methods**  **RCT**  Co-design of text messages  Survey | Over wight/ obesity | Adolescent (Individual) |  |  |  |  |  |  | Y |  |  | Behavioural | NR | NR | NR | NR |  |  |  |  |  |  |
| Perry and Hoffman, 2010, USA (82) | **MIXED**  Community advisory board (1 youth taking part)  Study planning Surveys  Focus groups | Obesity | Local community |  |  |  |  |  |  | Y |  |  | Participatory- CBPR | NR | NR | NR | NR |  |  |  |  |  |  |
| Power et al, 2010, USA (83)  Bindler et al, 2011, USA (84)  Steele et al, 2011, USA (85)  Wordell et al, 2012, USA (86) | **MIXED**  Focus groups  Peer mentoring component  Questionnaires | Obesity | Institution (secondary education) |  |  |  |  |  | Y |  |  |  | Multi/complex- theory (incl. SCT) |  | NR | NR | NR |  |  |  |  |  |  |
| Renzaho et al, 2015, Australia (87) | **Qualitative**  Discussions  Interviews  Focus groups | Obesity | Local community |  |  |  |  |  | Y |  |  |  | Multi-theory (incl. participatory) | NR | NR | NR | NR |  |  |  |  |  |  |
| Robbins et al, 2003, USA (88)  Robbins et al, 2006, USA (89)  Pfeiffer et al, 2019, USA (90) | **Qualitative**  **RCT**  Questionnaire Focus groups | Overweight/ Obesity | Institution (secondary education) |  |  |  |  |  | Y |  |  |  | Complex model (Transtheoretical model) | NR | NR | NR | NR |  |  |  |  |  |  |
| Martin Romero & Francis, 2020, USA (91) | **Qualitative,**  Focus groups  Discussions  Participatory activities | Obesity | Family |  |  |  |  |  | Y |  |  |  | Multi-theory (incl. Participatory + ecological) | NR | NR | NR | NR |  |  |  |  |  |  |
| Briancon et al, 2010, France (92)  Saez et al, 2018, France (93) | **RCT**  Peer mentors/facilitators  Focus groups | Overweight/obesity | Institution (secondary education) |  |  |  |  |  |  |  | Y |  | SCT | NR | NR | NR | NR |  |  |  |  |  |  |
| Gittelsohn et al, 2014, USA (94)  Sato et al, 2016, USA (95)  Ruggiero et al, 2018, USA (96) | **RCT + Qualitative**  Interviews, Focus groups,  Youth leaders | Obesity | Local community |  |  |  |  |  |  | Y |  |  | Multi-theory (incl. SCT) | NR | NR | NR | NR |  |  |  |  |  |  |
| Schwarte et al, 2010, USA (97) | **Qualitative**  Photovoice | Obesity | Local community |  |  |  |  |  |  | Y |  |  | Complex model | NR | NR | NR | NR |  |  |  |  |  |  |
| Shepherd et al, 2006, USA (98) | **Qualitative**  Interviews | Obesity | Adolescent (individual) |  |  |  |  |  | Y |  |  |  | NR | NR | NR | NR | NR |  |  |  |  |  |  |
| Sherman et al, 2011, Canada (99) | **Qualitative**  Focused ethnography  Interviews  Focus groups  Educational play | Type 2 diabetes | Local community |  |  |  |  |  | Y |  |  |  | Multi/complex-model (incl. participatory) | NR | NR | NR | NR |  |  |  |  |  |  |
| Spaulding et al, 2015, USA (100) | **Mixed**  Discussions  Focus groups | Overweight | Institution (secondary education) |  |  |  |  |  | Y |  |  |  | NR |  |  |  |  |  |  |  |  |  |  |
| Swanson et al, 2013, USA (101) | **Qualitative**  Focus groups  Interviews  Co-design | Overweight/ obesity | Local community |  |  |  |  |  | Y |  |  |  | Ecological model | NR | NR | NR | BR |  |  |  |  |  |  |
| Thompson et al, 2012, USA (102)  Cullen et al, 2013, USA (103) | **Qualitative**  **RCT**  Focus groups  Interviews  Co-design  Questionnaires | Obesity | Adolescent (individual) |  |  |  |  |  |  | Y |  |  | SCT | NR | NR | NR | NR |  |  |  |  |  |  |
| Swinburn et al, 2007 (Aust, NZ, Fiji, Tonga) (104)  Schultz et al, 2007 (Fiji) (105)  Utter et al, 2010, NZ (106)  Mattews et al, 2010, Aust (107)  Millar et al, 2011, Australia (108)  Swinburn et al, 2011 Australia (109)  Fotu et al, 2011, Fiji (110) | **MIXED**  Interviews Questionnaire and Workshop development  Focus groups | Obesity | Local community  &  Institution (secondary education) |  |  |  |  |  |  | Y |  |  | SCT | NR | NR | NR | NR |  |  |  |  |  |  |
| Vangeepuram et al, 2015, USA (111)  Vangeepuram et al, 2017, USA (112) | **RCT**  Peer-led  Workshops  Interviews  Focus groups | Type 2 diabetes | Local community |  |  |  |  |  |  | Y |  |  | Multi-theory (incl. participatory- CBPR & SCT) | NR | NR | NR | NR |  |  |  |  |  |  |
| Woodgate & Sigurdson 2015, Canada (113) | **Mixed**  Focus groups  Interviews  Health promotion activities | Cardiovascular health | Institution (secondary education) |  |  |  |  |  |  |  | Y |  | SCT | NR | NR | NR | NR |  |  |  |  |  |  |
| Werkhoven et al, 2018, Australia (114) | **Qualitative**  Questionnaire | Obesity | Adolescent (Individual-in a tertiary context) |  |  |  |  |  | Y |  |  |  | Complex model (Health at Every Size (HAES)  Fitness not Fatness) | NR | NR | NR | NR |  |  |  |  |  |  |
| Kattelmann et al, 2014, USA (115)  Kattelmann et al, 2014, USA (116) | **RCT + Qualitative**  Steering committee included young people.  Focus groups and interviews | Over-weight/ Obesity | Institution (tertiary) |  |  |  |  |  | Y |  |  |  | Multi/complex models (incl. participatory-CBPR) | NR | NR | NR | NR |  |  |  |  |  |  |
| Woodgate et al, 2010, Canada (117) | **Qualitative**  Ethnographic methods  interviews  Field work discussions  Photovoice | Determinants of health | Local community  &  Adolescent |  |  |  |  |  | Y |  |  |  | NR | NR | NR | NR | NR |  |  |  |  |  |  |
| Shah et al, 2011, Australia (118)  Shah et al, 2017, Australia (119)  Foley et al, 2017, Australia (120) | **Qualitative**  Peer leaders  Questionnaires | Obesity | Institution (secondary education) |  |  |  |  |  | Y |  |  |  | Multi/complex theory- (incl. SCT) | NR | NR | NR | NR |  |  |  |  |  |  |
| Surkan, et al, 2010, USA(121)  Dennisuk et al, 2011, USA (122)  Gittelsohn et al, 2013, USA (123)  Shin et al, 2015, USA (124) | **RCT**  Cross-sectional study  Questionnaires  Interviews  Peer leaders/ Peer educators | Obesity | Local community |  |  |  |  |  | Y |  |  |  | Social cognitive theory | NR | NR | NR | NR |  |  |  |  |  |  |
| Lane et al, 2019, USA (125) | **RCT + Qualitative**  Youth ambassadors/ leaders  Advocacy activities  Interviews/ focus groups. | Obesity | Local community |  |  |  |  |  |  | Y |  |  | Multi-theory (incl. Participatory + behavioural) |  | NR | NR | NR |  |  |  |  |  |  |
| Lems et al, 2019, The Netherlands (126) | **Qualitative**  Interviews  Co-creation sessions | Over-weight/ obesity | Local community |  |  |  |  |  |  | Y |  |  | Participatory- PAR |  | NR | NR | NR |  |  |  |  |  |  |

**Abbreviations:**

Y: Yes

Incl.: including

NR: Not Reported

RCT: Randomised Control Trial

YPAR: Youth Participatory Action Research

CBPR: Community Based Participatory Research

SCT: Social Cognitive Theory

**REFERENCES**

1. Anselma M, Chinapaw MJM, Altenburg TM. Determinants of Child Health Behaviors in a Disadvantaged Area from a Community Perspective: A Participatory Needs Assessment. International Journal of Environmental Research & Public Health [Electronic Resource]. 2018;15(4):31.

2. Anselma M, Altenburg T, Chinapaw M. Kids in Action: the protocol of a Youth Participatory Action Research project to promote physical activity and dietary behaviour. BMJ Open. 2019;9(3):e025584.

3. Anselma M, Altenburg TM, Emke H, van Nassau F, Jurg M, Ruiter RAC, et al. Co-designing obesity prevention interventions together with children: intervention mapping meets youth-led participatory action research. The international journal of behavioral nutrition and physical activity. 2019;16(1):130.

4. Anselma M, Chinapaw M, Altenburg T. "Not Only Adults Can Make Good Decisions, We as Children Can Do That as Well" Evaluating the Process of the Youth-Led Participatory Action Research 'Kids in Action'. International journal of environmental research and public health. 2020;17(2):625.

5. Arlinghaus KR, Moreno JP, Reesor L, Hernandez DC, Johnston CA. Companeros: High School Students Mentor Middle School Students to Address Obesity Among Hispanic Adolescents. Preventing Chronic Disease. 2017;14:E92.

6. Bailey J, Davies C, McCrossin T, Kiernan M, Skinner R, Steinbeck K, et al. Fit4YAMs: Structuring a Lifestyle Intervention for Rural Overweight and Obese Young Adult Males Using Participatory Design. Journal of Adolescent Health. 2018;62(3S):S65-S71.

7. Balvanz P, Dodgen L, Quinn J, Holloway T, Hudspeth S, Eng E. From Voice to Choice: African American Youth Examine Childhood Obesity in Rural North Carolina. Progress in Community Health Partnerships. 2016;10(2):293-303.

8. Bardwell G, Morton C, Chester A, Pancoska P, Buch S, Cecchetti A, et al. Feasibility of adolescents to conduct community-based participatory research on obesity and diabetes in rural Appalachia. Clinical and translational science. 2009;2(5):340-9.

9. Uyeda K, Bogart LM, Hawes-Dawson J, Schuster MA. Development and implementation of a school-based obesity prevention intervention: lessons learned from community-based participatory research. Progress in Community Health Partnerships. 2009;3(3):249-55.

10. Goh Y, Bogart LM, Sipple-Asher BK, Uyeda K, Hawes-Dawson J, Olarita-Dhungana J, et al. Using community-based participatory research to identify potential interventions to overcome barriers to adolescents' healthy eating and physical activity. Journal of Behavioral Medicine. 2009;32(5):491-502.

11. Bogart LM, Elliott MN, Uyeda K, Hawes-Dawson J, Klein DJ, Schuster MA. Preliminary healthy eating outcomes of SNaX, a pilot community-based intervention for adolescents. Journal of Adolescent Health. 2011;48(2):196-202.

12. Bogart LM, Cowgill BO, Elliott MN, Klein DJ, Hawes-Dawson J, Uyeda K, et al. A randomized controlled trial of Students for Nutrition and exercise: A community-based participatory research study. Journal of Adolescent Health. 2014;55(3):415-22.

13. Bogart LM, Elliott MN, Cowgill BO, Klein DJ, Hawes-Dawson J, Uyeda K, et al. Two-Year BMI Outcomes From a School-Based Intervention for Nutrition and Exercise: A Randomized Trial. Pediatrics. 2016;137(5).

14. Bogart LM, Fu CM, Eyraud J, Cowgill BO, Hawes-Dawson J, Uyeda K, et al. Evaluation of the dissemination of SNaX, a middle school-based obesity prevention intervention, within a large US school district. Transl Behav Med. 2018;8(5):724-32.

15. Christianson J, Kattelmann K, Riggsbee K, Moret L, Vilaro MJ, Olfert MD, et al. Promoting Wellness on College Campuses: Identifying and Addressing the Wellness Needs of College Students. Topics in Clinical Nutrition. 2019;34(2):125-37.

16. Chae SM, Yeo JY, Hwang JH, Lee JH, Lim J, Kwon I. Weight Control in Adolescents: Focus Groups With Korean Adolescents and Their Teachers. Journal of pediatric nursing. 2017;33:4-9.

17. Cassidy O, Sbrocco T, Vannucci A, Nelson B, Jackson-Bowen D, Heimdal J, et al. Adapting interpersonal psychotherapy for the prevention of excessive weight gain in rural African American girls. Journal of Pediatric Psychology. 2013;38(9):965-77.

18. Brown BD, Harris KJ, Harris JL, Parker M, Ricci C, Noonan C. Translating the Diabetes Prevention Program for Northern Plains Indian youth through community-based participatory research methods. Diabetes Educator. 2010;36(6):924-35.

19. Brown B, Noonan C, Harris KJ, Parker M, Gaskill S, Ricci C, et al. Developing and piloting the Journey to Native Youth Health program in Northern Plains Indian communities. Diabetes Educ. 2013;39(1):109-18.

20. Diez-Canseco F, Boeren Y, Quispe R, Chiang ML, Miranda JJ. Engagement of adolescents in a health communications program to prevent noncommunicable diseases: Multiplicadores Jovenes, Lima, Peru, 2011. Preventing Chronic Disease. 2015;12:E28.

21. Correa N, Rajaraman D, Swaminathan S, Vaz M, Jayachitra KG, Lear SA, et al. Perceptions of healthy eating amongst Indian adolescents in India and Canada. Appetite. 2017;116:471-9.

22. Rajaraman D, Correa N, Punthakee Z, Lear SA, Jayachitra KG, Vaz M, et al. Perceived Benefits, Facilitators, Disadvantages, and Barriers for Physical Activity Amongst South Asian Adolescents in India and Canada. 2015;12(7):931.

23. Cueva K, Speakman K, Neault N, Richards J, Lovato V, Parker S, et al. Cultural Connectedness as Obesity Prevention: Indigenous Youth Perspectives on Feast for the Future. Journal of nutrition education and behavior. 2020;07.

24. Debar LL, Schneider M, Drews KL, Ford EG, Stadler DD, Moe EL, et al. Student public commitment in a school-based diabetes prevention project: Impact on physical health and health behavior. BMC Public Health. 2011;11.

25. DeBar LL, Schneider M, Ford EG, Hernandez AE, Showell B, Drews KL, et al. Social marketing-based communications to integrate and support the HEALTHY study intervention. International journal of obesity (2005). 2009;33 Suppl 4(Suppl 4):S52-S9.

26. Group HS, Hirst K, Baranowski T, DeBar L, Foster GD, Kaufman F, et al. HEALTHY study rationale, design and methods: moderating risk of type 2 diabetes in multi-ethnic middle school students. International journal of obesity. 2005;33(4).

27. Group HS, Hirst K, Baranowski T, DeBar L, Foster GD, Kaufman F, et al. HEALTHY study rationale, design and methods: moderating risk of type 2 diabetes in multi-ethnic middle school students. International Journal of Obesity. 2009;33 Suppl 4:S4-20.

28. Siega-Riz AM, El Ghormli L, Mobley C, Gillis B, Stadler D, Hartstein J, et al. The effects of the HEALTHY study intervention on middle school student dietary intakes. International Journal of Behavioral Nutrition and Physical Activity. 2011;8(1):7.

29. Venditti EM, Elliot DL, Faith MS, Firrell LS, Giles CM, Goldberg L, et al. Rationale, design and methods of the HEALTHY study behavior intervention component. International journal of obesity. 2005;33(4).

30. Venditti EM, Elliot DL, Faith MS, Firrell LS, Giles CM, Goldberg L, et al. Rationale, design and methods of the HEALTHY study behavior intervention component. International Journal of Obesity. 2009;33 Suppl 4:S44-51.

31. Marcus MD, Hirst K, Kaufman F, Foster GD, Baranowski T. Lessons learned from the HEALTHY primary prevention trial of risk factors for type 2 diabetes in middle school youth. Curr Diab Rep. 2013;13(1):63-71.

32. de Vet E, de Wit JB, Luszczynska A, Stok FM, Gaspar T, Pratt M, et al. Access to excess: how do adolescents deal with unhealthy foods in their environment? European journal of public health. 2013;23(5):752-6.

33. Edwardson CL, Harrington DM, Yates T, Bodicoat DH, Khunti K, Gorely T, et al. A cluster randomised controlled trial to investigate the effectiveness and cost effectiveness of the 'Girls Active' intervention: a study protocol. BMC Public Health. 2015;15:526.

34. Harrington DM, Davies MJ, Bodicoat DH, Charles JM, Chudasama YV, Gorely T, et al. Effectiveness of the 'Girls Active' school-based physical activity programme: A cluster randomised controlled trial. Int J Behav Nutr Phys Act. 2018;15(1):40.

35. Ewan L, McLinden D, Biro F, DeJonckheere M, Vaughn L. Mapping the views of adolescent health stakeholders. Journal of Adolescent Health. 2015;56(2):S68-S9.

36. Findholt NE, Michael YL, Davis MM. Photovoice engages rural youth in childhood obesity prevention. Public Health Nursing. 2011;28(2):186-92.

37. Frerichs L, Brittin J, Stewart C, Robbins R, Riggs C, Mayberger S, et al. SaludableOmaha: development of a youth advocacy initiative to increase community readiness for obesity prevention, 2011-2012. Preventing Chronic Disease. 2012;9:E173.

38. Frerichs L, Brittin J, Robbins R, Steenson S, Stewart C, Fisher C, et al. SaludABLEOmaha: improving readiness to address obesity through healthy lifestyle in a Midwestern Latino Community, 2011-2013. Preventing Chronic Disease. 2015;12(2).

39. Frerichs L, Hassmiller Lich K, Young TL, Dave G, Stith D, Corbie-Smith G. Development of a Systems Science Curriculum to Engage Rural African American Teens in Understanding and Addressing Childhood Obesity Prevention. Health Education & Behavior. 2018;45(3):423-34.

40. Garcia ML, Gatdula N, Bonilla E, Frank GC, Bird M, Rascon MS, et al. Engaging Intergenerational Hispanics/Latinos to Examine Factors Influencing Childhood Obesity Using the PRECEDE-PROCEED Model. Maternal & Child Health Journal. 2019;23(6):802-10.

41. Garnett BR, Wendel J, Banks C, Goodridge A, Harding R, Harris R, et al. Challenges of Data Dissemination Efforts Within a Community-Based Participatory Project About Persistent Racial Disparities in Excess Weight. Progress in community health partnerships : research, education, and action. 2015;9(2):289-98.

42. Green B, Ralston PA, Young-Clark I, Waryoba C, Smith S, Harris CM, et al. A Youth Health Leadership Program: Feasibility and Initial Outcomes. Journal of community health. 2019;07.

43. Hagedorn RL, White JA, Franzen-Castle L, Colby SE, Kattelmann KK, White AA, et al. Teens Implementing a Childhood Obesity Prevention Program in the Community: Feasibility and Perceptions of a Partnership with HSTA and iCook 4-H. International Journal of Environmental Research & Public Health [Electronic Resource]. 2018;15(5):07.

44. Grignard S, Bourguignon JP, Born M, Mairiaux P, Vandoorne C. Characteristics of adolescent attempts to manage overweight. Patient Education & Counseling. 2003;51(2):183-9.

45. Hannay J, Dudley R, Milan S, Leibovitz PK. Combining Photovoice and focus groups: engaging Latina teens in community assessment. American Journal of Preventive Medicine. 2013;44(3 Suppl 3):S215-24.

46. Jackson CJ, Mullis RM, Hughes M. Development of a theater-based nutrition and physical activity intervention for low-income, urban, African American adolescents. Progress in community health partnerships : research, education, and action. 2010;4(2):89-98.

47. Hunsberger M, McGinnis P, Smith J, Beamer BA, O'Malley J. Calorie labeling in a rural middle school influences food selection: findings from community-based participatory research. Journal of Obesity. 2015;2015:531690.

48. Knoblock-Hahn AL, Wray R, LeRouge CM. Perceptions of Adolescents with Overweight and Obesity for the Development of User-Centered Design Self-Management Tools within the Context of the Chronic Care Model: A Qualitative Study. Journal of the Academy of Nutrition & Dietetics. 2016;116(6):957-67.

49. LeRouge C, Durneva P, Sangameswaran S, Gloster A-M. Design Guidelines for a Technology-Enabled Nutrition Education Program to Support Overweight and Obese Adolescents: Qualitative User-Centered Design Study. Journal of medical Internet research. 2019;21(7):e14430-e.

50. LeRouge CM, Hah H, Deckard GJ, Jiang H. Designing for the Co-Use of Consumer Health Technology in Self-Management of Adolescent Overweight and Obesity: Mixed Methods Qualitative Study. JMIR Mhealth Uhealth. 2020;8(6):e18391.

51. Kong AS, Farnsworth S, Canaca JA, Harris A, Palley G, Sussman AL. An Adaptive Community-Based Participatory Approach to Formative Assessment With High Schools for Obesity Intervention. Journal of School Health. 2012;82(3):147-54.

52. Sussman AL, Montoya C, Werder O, Davis S, Wallerstein N, Kong AS. An adaptive CBPR approach to create weight management materials for a school-based health center intervention. Journal of obesity. 2013;2013:978482-.

53. Kramer L, Schwartz P, Cheadle A, Borton JE, Wright M, Chase C, et al. Promoting Policy and Environmental Change Using Photovoice in the Kaiser Permanente Community Health Initiative. Health Promotion Practice. 2010;11(3):332-9.

54. Kramer L, Schwartz P, Cheadle A, Rauzon S. Using photovoice as a participatory evaluation tool in Kaiser Permanente's Community Health Initiative. Health Promotion Practice. 2013;14(5):686-94.

55. Lane HG, Driessen R, Campbell K, Deitch R, Turner L, Parker EA, et al. Development of the PEA-PODS (Perceptions of the Environment and Patterns of Diet at School) survey for students. Preventing Chronic Disease. 2018;15(6).

56. Lewis RK, Lee FA, Brown KK, LoCurto J, Stowell D, Maryman J, et al. Youth empowerment implementation project evaluation results: A program designed to improve the health and well-being of low-income African-American adolescents. Journal of prevention & intervention in the community. 2018;46(1):28-42.

57. Linton LS, Edwards CC, Woodruff SI, Millstein EA, Moder C. Youth advocacy as a tool for environmental and policy changes that support physical activity and nutrition: An evaluation study in San Diego County. Preventing Chronic Disease. 2014;11(3).

58. Millstein RA, Woodruff SI, Linton LS, Edwards CC, Sallis JF. Development of measures to evaluate youth advocacy for obesity prevention. International Journal of Behavioral Nutrition & Physical Activity. 2016;13:84.

59. Livingood WC, Monticalvo D, Bernhardt JM, Wells KT, Harris T, Kee K, et al. Engaging Adolescents Through Participatory and Qualitative Research Methods to Develop a Digital Communication Intervention to Reduce Adolescent Obesity. Health Education & Behavior. 2017;44(4):570-80.

60. Llaurado E, Aceves-Martins M, Tarro L, Papell-Garcia I, Puiggros F, Arola L, et al. A youth-led social marketing intervention to encourage healthy lifestyles, the EYTO (European Youth Tackling Obesity) project: a cluster randomised controlled trial in Catalonia, Spain. BMC Public Health. 2015;15(607).

61. Tarro L, Aceves-Martins M, Papell-Garcia I, Arola L, Giralt M, Llauradó E, et al. A youth-led, social marketing intervention run by adolescents to encourage healthy lifestyles among younger school peers (EYTO-Kids Project): a protocol for pilot cluster randomized controlled trial (Spain). International Journal of Environmental Research and Public Health. 2017;14(8):923.

62. Aceves-Martins M, Llauradó E, Tarro L, Moriña D, Papell-Garcia I, Prades-Tena J, et al. A School-Based, Peer-Led, Social Marketing Intervention To Engage Spanish Adolescents in a Healthy Lifestyle ("We Are Cool"--Som la Pera Study): A Parallel-Cluster Randomized Controlled Study. Childhood Obesity. 2017;13(4):300-13.

63. Tarro L, Llauradó E, Aceves-Martins M, Moriña D, Papell-Garcia I, Arola L, et al. Impact of a youth-led social marketing intervention run by adolescents to encourage healthy lifestyles among younger school peers (EYTO-Kids project): a parallel-cluster randomised controlled pilot study. J Epidemiol Community Health. 2019;73(4):324-33.

64. Llauradó E, Aceves-Martins M, Tarro L, Papell-Garcia I, Puiggròs F, Prades-Tena J, et al. The "Som la Pera" intervention: sustainability capacity evaluation of a peer-led social-marketing intervention to encourage healthy lifestyles among adolescents. Transl Behav Med. 2018;8(5):739-44.

65. Loman DG. Promoting physical activity in teen girls: insight from focus groups. Mcn. 2008;The American journal of maternal child nursing. 33(5):294-9; quiz 300-1.

66. Mauriello LM, Driskell MM, Sherman KJ, Johnson SS, Prochaska JM, Prochaska JO. Acceptability of a school-based intervention for the prevention of adolescent obesity. Journal of School Nursing. 2006;22(5):269-77.

67. Mauriello LM, Ciavatta MMH, Paiva AL, Sherman KJ, Castle PH, Johnson JL, et al. Results of a multi-media multiple behavior obesity prevention program for adolescents. Preventive medicine. 2010;51(6):451-6.

68. Maynard MJ, Baker G, Rawlins E, Anderson A, Harding S. Developing obesity prevention interventions among minority ethnic children in schools and places of worship: The DEAL (DiEt and Active Living) study. BMC Public Health. 2009;9:480.

69. Rawlins E, Baker G, Maynard M, Harding S. Perceptions of healthy eating and physical activity in an ethnically diverse sample of young children and their parents: the DEAL prevention of obesity study. Journal of Human Nutrition & Dietetics. 2013;26(2):132-44.

70. Maynard M, Baker G, Harding S. Exploring childhood obesity prevention among diverse ethnic groups in schools and places of worship: Recruitment, acceptability and feasibility of data collection and intervention components. Preventive Medicine Reports. 2017;6:130-6.

71. McGee BB, Richardson V, Johnson G, Johnson C. Perceptions of Food Intake, Physical Activity, and Obesity Among African-American Children in the Lower Mississippi Delta. American Journal of Health Promotion. 2017;31(4):333-5.

72. McKinney C, Bishop V, Cabrera K, Medina R, Takawira D, Donate N, et al. NuFit: nutrition and fitness CBPR program evaluation. Journal of Prevention & Intervention in the Community. 2014;42(2):112-24.

73. McPherson AC, Lindsay S. How do children with disabilities view 'healthy living'? A descriptive pilot study. Disability & Health Journal. 2012;5(3):201-9.

74. Morales-Campos DY, Parra-Medina D, Esparza LA. Picture this!: using participatory photo mapping with Hispanic girls. Family & Community Health. 2015;38(1):44-54.

75. Mosavel M, Gough MZ, Ferrell D. Using Asset Mapping to Engage Youth in Community-Based Participatory Research: The WE Project. Progress in Community Health Partnerships. 2018;12(2):223-36.

76. Muturi N, Kidd T, Daniels AM, Kattelmann KK, Khan T, Lindshield E, et al. Examining the role of youth empowerment in preventing adolescence obesity in low-income communities. Journal of Adolescence. 2018;68:242-51.

77. Necheles JW, Chung EQ, Hawes-Dawson J, Ryan GW, Williams SB, Holmes HN, et al. The Teen Photovoice Project: a pilot study to promote health through advocacy. Progress in Community Health Partnerships. 2007;1(3):221-9.

78. Nguyen NM, Dibley MJ, Tang HK, Alam A. Perceptions and Practices Related to Obesity in Adolescent Students and Their Programmatic Implications: Qualitative Evidence from Ho Chi Minh City, Vietnam. Maternal and Child Health Journal. 2017;21(12):2199-208.

79. Tang HK, Nguyen N-M, Dibley MJ, Nguyen THHD, Alam A. Improving the Lifestyle of Adolescents Through Peer Education and Support in Vietnam: Protocol for a Pilot Cluster Randomized Controlled Trial. JMIR Res Protoc. 2020;9(6):e15930-e.

80. Partridge SR, Raeside R, Latham Z, Singleton AC, Hyun K, Grunseit A, et al. 'Not to be harsh but try less to relate to 'the teens and you'll relate to them more': Co-designing obesity prevention text messages with adolescents. International Journal of Environmental Research and Public Health. 2019;16(24).

81. Partridge SR, Raeside R, Singleton AC, Hyun K, Latham Z, Grunseit A, et al. Text Message Behavioral Intervention for Teens on Eating, Physical Activity and Social Wellbeing (TEXTBITES): Protocol for a Randomized Controlled Trial. JMIR Res Protoc. 2020;9(2):e16481.

82. Perry C, Hoffman B. Assessing tribal youth physical activity and programming using a community-based participatory research approach. Public Health Nursing. 2010;27(2):104-14.

83. Power TG, Bindler RC, Goetz S, Daratha KB. Obesity prevention in early adolescence: Student, parent, and teacher views. Journal of School Health. 2010;80(1):13-9.

84. Bindler RC, Goetz S, Butkus SN, Power TG, Ullrich-French S, Steele M. The Process of Curriculum Development and Implementation for an Adolescent Health Project in Middle Schools. The Journal of School Nursing. 2011;28(1):13-23.

85. Steele MM, Daratha KB, Bindler RC, Power TG. The relationship between self-efficacy for behaviors that promote healthy weight and clinical indicators of adiposity in a sample of early adolescents. Health Educ Behav. 2011;38(6):596-602.

86. Wordell D, Daratha K, Mandal B, Bindler R, Butkus SN. Changes in a Middle School Food Environment Affect Food Behavior and Food Choices. Journal of the Academy of Nutrition and Dietetics. 2012;112(1):137-41.

87. Renzaho AM, Halliday JA, Mellor D, Green J. The Healthy Migrant Families Initiative: development of a culturally competent obesity prevention intervention for African migrants. BMC public health. 2015;15:272.

88. Robbins LB, Pender NJ, Kazanis AS. Barriers to physical activity perceived by adolescent girls. Journal of Midwifery & Women's Health. 2003;48(3):206-12.

89. Robbins LB, Gretebeck KA, Kazanis AS, Pender NJ. Girls on the Move Program to Increase Physical Activity Participation. Nursing Research. 2006;55(3):206-16.

90. Pfeiffer KA, Robbins LB, Ling J, Sharma DB, Dalimonte-Merckling DM, Voskuil VR, et al. Effects of the Girls on the Move randomized trial on adiposity and aerobic performance (secondary outcomes) in low-income adolescent girls. Pediatr Obes. 2019;14(11):e12559.

91. Martin Romero MY, Francis LA. Youth involvement in food preparation practices at home: A multi-method exploration of Latinx youth experiences and perspectives. Appetite. 2020;144 (no pagination).

92. Briançon S, Bonsergent E, Agrinier N, Tessier S, Legrand K, Lecomte E, et al. PRALIMAP: study protocol for a high school-based, factorial cluster randomised interventional trial of three overweight and obesity prevention strategies. Trials. 2010;11:119.

93. Saez L, Legrand K, Alleyrat C, Ramisasoa S, Langlois J, Muller L, et al. Using facilitator-receiver peer dyads matched according to socioeconomic status to promote behaviour change in overweight adolescents: a feasibility study. BMJ Open. 2018;8(6):e019731.

94. Gittelsohn J, Anderson Steeves E, Mui Y, Kharmats AY, Hopkins LC, Dennis D. B'More Healthy Communities for Kids: design of a multi-level intervention for obesity prevention for low-income African American children. BMC Public Health. 2014;14:942.

95. Sato PM, Steeves EA, Carnell S, Cheskin LJ, Trude AC, Shipley C, et al. A youth mentor-led nutritional intervention in urban recreation centers: a promising strategy for childhood obesity prevention in low-income neighborhoods. Health Education Research. 2016;31(2):195-206.

96. Ruggiero CF, Poirier L, Trude ACB, Yang T, Schwendler T, Gunen B, et al. Implementation of B'More Healthy Communities for Kids: process evaluation of a multi-level, multi-component obesity prevention intervention. Health education research. 2018;33(6):458-72.

97. Schwarte L, Samuels SE, Capitman J, Ruwe M, Boyle M, Flores G. The Central California Regional Obesity Prevention Program: changing nutrition and physical activity environments in California's heartland. American Journal of Public Health. 2010;100(11):2124-8.

98. Shepherd LM, Neumark-Sztainer D, Beyer KM, Story M. Should we discuss weight and calories in adolescent obesity prevention and weight-management programs? Perspectives of adolescent girls. Journal of the American Dietetic Association. 2006;106(9):1454-8.

99. Sherman J, Macdonald ME, Carnevale F, Vignola S. The development and implementation of a type 2 diabetes prevention program for youth in the Algonquin community of Rapid Lake, Quebec. Pimatisiwin A Journal of Aboriginal and Indigenous Community Health. 2011;9(1):219-44.

100. Spaulding CJ, McNeal CJ, Coppin JD, Shimek C, Field L, Murano PS. Engaging inner city middle school students in development of an energy expenditure food label. Journal of Prevention & Intervention in the Community. 2015;43(2):123-34.

101. Swanson M, Schoenberg NE, Davis R, Wright S, Dollarhide K. Perceptions of Healthful Eating and Influences on the Food Choices of Appalachian Youth. Journal of Nutrition Education and Behavior. 2013;45(2):147-53.

102. Thompson D, Cullen KW, Boushey C, Konzelmann K. Design of a website on nutrition and physical activity for adolescents: Results from formative research. Journal of Medical Internet Research. 2012;14(2):85-99.

103. Cullen KW, Thompson D, Boushey C, Konzelmann K, Chen TA. Evaluation of a web-based program promoting healthy eating and physical activity for adolescents: teen choice: food and fitness. Health Educ Res. 2013;28(4):704-14.

104. Swinburn B, Pryor J, McCabe M, Carter R, de Courten M, Schaaf D, et al. The Pacific OPIC Project (Obesity Prevention in Communities) -- objectives and designs. Pac Health Dialog. 2007;14(2):139-46.

105. Schultz J, Utter J, Mathews L, Cama T, Mavoa H, Swinburn B. The Pacific OPIC Project (Obesity Prevention in Communities): action plans and interventions. Pac Health Dialog. 2007;14(2):147-53.

106. Utter J, Warbrick J, Scragg R, Denny S, Schaaf D. Design, development, and achievements of a youth-led nutrition and physical activity intervention in a Pacific community in New Zealand. Journal of the American Dietetic Association. 2010;110(11):1634-7.

107. Mathews LB, Moodie MM, Simmons AM, Swinburn BA. The process evaluation of It's Your Move!, an Australian adolescent community-based obesity prevention project. BMC Public Health. 2010;10:448.

108. Millar L, Kremer P, de Silva-Sanigorski A, McCabe MP, Mavoa H, Moodie M, et al. Reduction in overweight and obesity from a 3-year community-based intervention in Australia: the 'It's Your Move!' project. Obes Rev. 2011;12 Suppl 2:20-8.

109. Swinburn BA, Millar L, Utter J, Kremer P, Moodie M, Mavoa H, et al. The Pacific Obesity Prevention in Communities project: project overview and methods. Obes Rev. 2011;12 Suppl 2:3-11.

110. Fotu KF, Moodie MM, Mavoa HM, Pomana S, Schultz JT, Swinburn BA. Process evaluation of a community-based adolescent obesity prevention project in Tonga. BMC Public Health. 2011;11(1):284.

111. Vangeepuram N, Carmona J, Arniella G, Horowitz CR, Burnet D. Use of Focus Groups to Inform a Youth Diabetes Prevention Model. J Nutr Educ Behav. 2015;47(6):532-9.e1.

112. Vangeepuram N, Williams N, Constable J, Waldman L, Lopez-Belin P, Phelps-Waldropt L, et al. TEEN HEED: Design of a clinical-community youth diabetes prevention intervention. Contemporary Clinical Trials. 2017;57:23-8.

113. Woodgate RL, Sigurdson CM. Building school-based cardiovascular health promotion capacity in youth: a mixed methods study. BMC Public Health. 2015;15:421.

114. Werkhoven T, Cotton W, Dudley D. Australian tertiary students' attitudes towards youth obesity in educational institutions. European Physical Education Review. 2018;24(2):181-93.

115. Kattelmann KK, White AA, Greene GW, Byrd-Bredbenner C, Hoerr SL, Horacek TM, et al. Development of Young Adults Eating and Active for Health (YEAH) Internet-Based Intervention via a Community-Based Participatory Research Model. Journal of Nutrition Education and Behavior. 2014;46(2):S10-S25.

116. Kattelmann KK, Bredbenner CB, White AA, Greene GW, Hoerr SL, Kidd T, et al. The Effects of Young Adults Eating and Active for Health (YEAH): A Theory-Based Web-Delivered Intervention. Journal of Nutrition Education and Behavior. 2014;46(6):S27-S41.

117. Woodgate RL, Leach J. Youth’s Perspectives on the Determinants of Health. Qualitative Health Research. 2010;20(9):1173-82.

118. Shah S, Patching van der Sluijs C, Lagleva M, Pesle A, Lim KS, Bittar H, et al. A partnership for health - working with schools to promote healthy lifestyle. Aust Fam Physician. 2011;40(12):1011-3.

119. Shah S, Foley BC, Molinari I, Lim KS, Shrewsbury VA. The Students As LifeStyle Activists (SALSA) program. Br J Sports Med. 2017;51(19):1445-6.

120. Foley BC, Shrewsbury VA, Hardy LL, Flood VM, Byth K, Shah S. Evaluation of a peer education program on student leaders' energy balance-related behaviors. BMC Public Health. 2017;17(1):695.

121. Surkan PJ, Coutinho AJ, Christiansen K, Dennisuk LA, Suratkar S, Mead E, et al. Healthy food purchasing among African American youth: associations with child gender, adult caregiver characteristics and the home food environment. Public Health Nutrition. 2011;14(4):670-7.

122. Dennisuk LA, Coutinho AJ, Suratkar S, Surkan PJ, Christiansen K, Riley M, et al. Food Expenditures and Food Purchasing Among Low-Income, Urban, African-American Youth. American Journal of Preventive Medicine. 2011;40(6):625-8.

123. Gittelsohn J, Dennisuk LA, Christiansen K, Bhimani R, Johnson A, Alexander E, et al. Development and implementation of Baltimore Healthy Eating Zones: a youth-targeted intervention to improve the urban food environment. Health Educ Res. 2013;28(4):732-44.

124. Shin A, Surkan PJ, Coutinho AJ, Suratkar SR, Campbell RK, Rowan M, et al. Impact of Baltimore Healthy Eating Zones: An Environmental Intervention to Improve Diet Among African American Youth. Health Education & Behavior. 2015;42(1):97S-105S.

125. Lane HG, Porter KJ, Hecht E, Harris P, Zoellner JM. A Participatory Process to Engage Appalachian Youth in Reducing Sugar-Sweetened Beverage Consumption. Health Promotion Practice. 2019;20(2):258-68.

126. Lems E, Hilverda F, Broerse JEW, Dedding C. 'Just stuff yourself': Identifying health-promotion strategies from the perspectives of adolescent boys from disadvantaged neighbourhoods. Health Expect. 2019;22(5):1040-9.
